# Metal-Free Homogeneous O_2_ Reduction by an Iminium-Based Electrocatalyst

**DOI:** 10.1021/jacs.3c14549

**Published:** 2024-03-15

**Authors:** Emma N. Cook, Anna E. Davis, Michael K. Hilinski, Charles W. Machan

**Affiliations:** Department of Chemistry, University of Virginia, PO Box 400319, Charlottesville, Virginia 22904-4319, United States

## Abstract

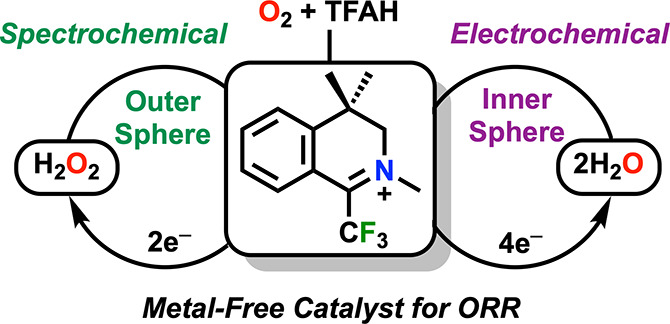

The oxygen reduction reaction (ORR) is important for alternative energy and industrial oxidation processes. Herein, an iminium-based organoelectrocatalyst (**im**^**+**^) for the ORR with trifluoroacetic acid as a proton source in acetonitrile solution under both electrochemical and spectrochemical conditions using decamethylferrocene as a chemical reductant is reported. Under spectrochemical conditions, H_2_O_2_ is the primary reaction product, while under electrochemical conditions H_2_O is produced. This difference in selectivity is attributed to the interception of the free superoxide intermediate under electrochemical conditions by the reduced catalyst, accessing an alternate inner-sphere pathway.

The increasing atmospheric carbon dioxide (CO_2_) concentration has had detrimental impacts on our environment and creates a drastic need for alternative energy processes. The oxygen reduction reaction (ORR) is important in fuel cells and alternative energy devices such as zinc-air batteries, in addition to a green alternative for H_2_O_2_ production.^1,2^ Researchers have focused on open-shell transition-metal complexes as catalysts for this reaction due to their often facile reactivity with the triplet ground state of dioxygen (O_2_). There has also been some advancement in the development of carbon-based catalysts for the ORR.^2−8^ Comparatively, the use of homogeneous organic molecules for catalytic ORR has been less widely studied since reduced oxygen species (ROSs) formed as intermediates (e.g., superoxide O_2_^•–^) can degrade organic molecules.

In 2020, Karimi *et al.* reported the ORR activity of carbenium dications using decamethylferrocene (Cp*_2_Fe) as a chemical reductant with methanesulfonic acid as a proton source (Figure 1).^9^ The reduced carbene radicals were found to rapidly react via an inner-sphere mechanism to form an intermediate peroxide, which is protonated to release H_2_O_2_. Recently, Tanjedrew *et al.*reported imidazole-benzimidazole electrocatalysts for ORR under aqueous conditions.^10^ They proposed that O_2_ binds to the reduced catalyst to form a superoxide species, which is further reduced and protonated to produce H_2_O_2_. Homogeneous organic species have also been shown to be active for the outer-sphere reduction of O_2_, with free O_2_^•–^ as an intermediate. Electrocatalytic ORR by an outer-sphere mechanism was first reported in 1985 by Andrieux et al. using methylviologen to generate H_2_O_2_ in acidic dimethylsulfoxide (DMSO).^11^ Outer-sphere electron transfer to generate O_2_^•–^ was followed by protonation to HO_2_^•^, which was subsequently reduced by the regenerated methyl viologen monocation to HO_2_^–^ and then protonated to form H_2_O_2_. Following this, a 1993 study by Audebert and Hapiot found that substituted 9-(4-X-phenyl)-*N*-methylacridinium salts in acidic DMSO also effectively reduce O_2_ to H_2_O_2_ via an analogous mechanism.^12^

Previously, 3,4-dihydro-2,4,4-trimethyl-1-(trifluoromethyl)isoquinolinium tetrafluoroborate (**im**^**+**^, Figure 1) was reported to be a hydroxylation catalyst with H_2_O_2_ as an oxidant.^13,14^ It was hypothesized that this iminium could mediate outer-sphere O_2_ reduction similar to related organic cations and that its intrinsic stability to H_2_O_2_ would be beneficial to catalyst stability.^15^ Catalytic and mechanistic experiments reveal that the iminium salt is an efficient catalyst for ORR to H_2_O and H_2_O_2_ in acetonitrile (MeCN) with trifluoroacetic acid (TFAH) as the proton source. Under spectrochemical conditions with Cp*_2_Fe as a chemical reductant in solution, O_2_ is quantitatively reduced via an outer-sphere mechanism to H_2_O_2_. Under electrochemical conditions, O_2_^•–^ is intercepted by the reduced iminium in solution, accessing an inner-sphere mechanism to quantitatively produce H_2_O. The difference in selectivity is proposed to be regulated by the simultaneous availability of O_2_^•–^ and the reduced iminium in higher concentrations in the reaction-diffusion layer.

**Figure 1:**
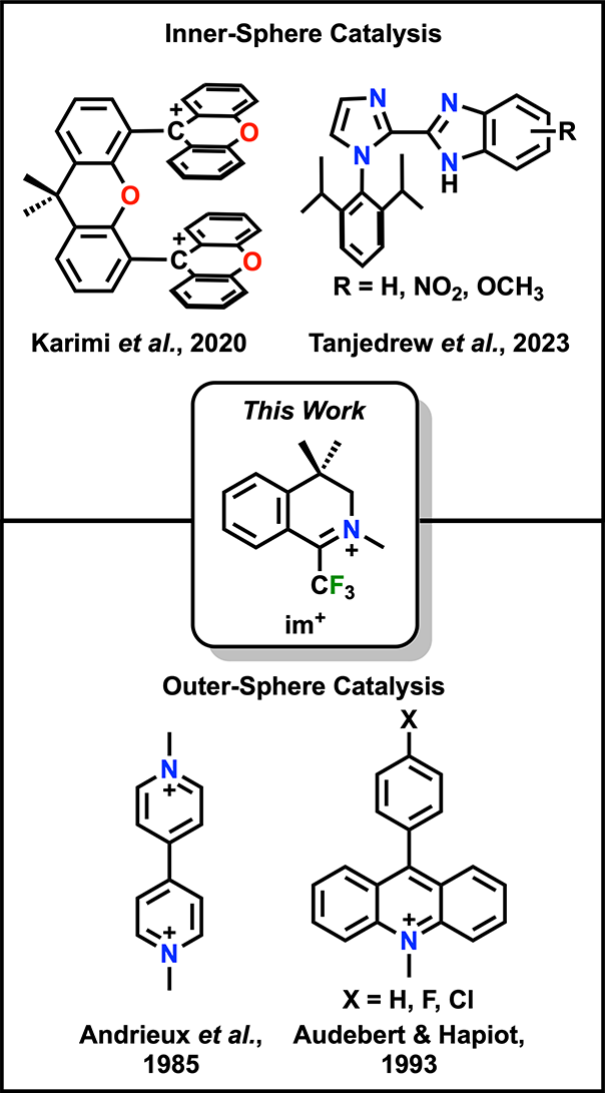
Summary of previously reported organic-based catalysts for the ORR and the catalyst (**im**^**+**^) described here.

**Im**^**+**^ was analyzed by cyclic voltammetry (CV) with tetrabutylammonium hexafluorophosphate (TBAPF_6_) as the supporting electrolyte in MeCN (Figure 2). Under Ar saturation conditions, there is a diffusion-limited irreversible reduction feature, which is attributed to the reduction of the iminium to a carbon-based radical species (**im**^**0**^, see below) at *E*_p_ = −0.82 V vs Fc^+^/Fc. Due to irreversibility at scan rates below 2 V/s, the *E*_1/2_ was estimated to be −0.77 V vs Fc^+^/Fc by taking the first derivative of the current density (Figures S1 & S5).^16^ This irreversible feature is consistent with a radical–radical dimerization (RRD) mechanism occurring between two equivalents of **im**^**0**^, as evidenced by an evaluation of peak potential dependence on both scan rate and concentration (Figures S1 & S2).^17^ Upon saturation of the solution with O_2_, there is an observed 150 mV positive potential shift of the irreversible reduction feature to *E*_p_ = −0.67 V vs Fc^+^/Fc, suggestive of a strong binding interaction. Interestingly, variable concentration and scan rate studies suggested a shift to a radical-substrate dimerization (RSD) mechanism, based on the observed dependence of *E*_p_ on scan rate and concentration under these conditions (Figures S3 & S4). This observation is proposed to correlate to a favorable interaction between **im**^**0**^ and O_2_^•–^ at reducing potentials.^17^ Addition of TFAH (p*K*_a_(MeCN) = 12.65)^18^ as a proton source under Ar saturation conditions resulted in an increase in current (Figures 2 & S5); the dependence of *E*_p_ on scan rate and concentration suggests an RSD mechanism, implying that a reaction with TFAH precedes the dimerization of the **im**^**0**^ radical (Figures S6 & S7). However, under O_2_ saturation in the presence of TFAH, a large increase in current density is again observed 150 mV positive of the Faradaic response, consistent with catalytic O_2_ reduction, implying that dimerization is not relevant under these conditions. A CV rinse test and control experiments demonstrated that the observed catalytic response of **im**^**+**^ is homogeneous (Figure S8).

**Figure 2:**
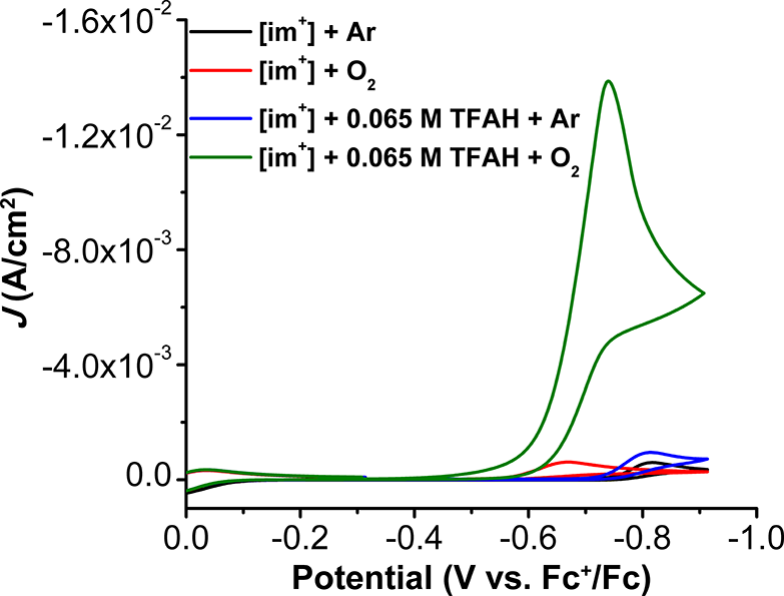
CVs of **im**^**+**^ under Ar and O_2_ saturation conditions with and without acid. Conditions: 1.3 mM **im**^**+**^, 0.1 M TBAPF_6_/MeCN; glassy carbon working electrode, glassy carbon counter electrode, Ag/AgCl pseudoreference electrode; 100 mV/s scan rate.

Independently varying the concentrations of **im**^**+**^, O_2_ and TFAH revealed a first-order concentration dependence on **im**^**+**^, O_2_, and TFAH in the current response (eq 1, Figures S9–S11). As the concentration of **im**^**+**^ decreases, there is an observed negative potential shift (Figure S9), which conforms to the behavior expected for an outer-sphere electron transfer followed by rapid catalytic reaction steps.^11^ As the concentration of O_2_ decreases, a decrease in the catalytic wave at *E*_p_ = −0.67 V vs Fc^+^/Fc is observed, as well as the recovery of the **im**^**+**^/**im**^**0**^ redox feature at *E*_p_ = −0.82 V vs Fc^+^/Fc. The reappearance of the intrinsic redox feature suggests the system is operating under total catalysis conditions, where O_2_ is rapidly consumed within the reaction-diffusion layer and excess **im**^**+**^ is available for reduction at more negative potentials. The recovery of the Faradaic redox response has also been observed for ORR mediated by methyl viologen and phenylacridinium salts, which are proposed to have an outer-sphere mechanism.^11,12^


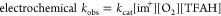
1Rotating ring-disk electrode (RRDE) methods with a glassy carbon disk and roughened gold ring^19^ were used to determine the selectivity of ORR by **im**^**+**^ under electrochemical conditions. Under air saturation, this system was found to be 92.6 ± 1.3% selective for H_2_O (see Supporting Information, Figures S14–S16). Control CV studies with added urea·H_2_O_2_ showed a slight decrease in current density (Figures S12 & S13) under Ar saturation conditions without a shift to positive potentials, which suggests a relatively slower reaction between **im**^**0**^ and H_2_O_2_. Consistent with this, under O_2_ saturation the catalytic current density is recovered, as is the shift to more positive potentials, confirming that **im**^**0**^ preferably reacts with O_2_ over H_2_O_2_.

Catalytic ORR activity of **im**^**+**^ was then studied by stopped-flow UV–vis methods using Cp*_2_Fe as a chemical reductant and TFAH as the proton source. The growth of the spectral handle of [Cp*_2_Fe]^+^ at 780 nm was monitored to extract the kinetic parameters for the reaction (Figure 3). The rate law under these conditions (eq 2) was determined by independently varying the concentration of **im**^**+**^, TFAH, O_2_, and Cp*_2_Fe (Figures S18–S21). These studies revealed that ORR shows a first-order concentration dependence on [**im**^**+**^] and is independent of TFAH, O_2_, and Cp*_2_Fe concentration, indicative of saturation kinetics at low catalyst concentration (4 μM). A Ti(O)SO_4_ colorimetric assay was used to determine the selectivity of ORR by **im**^**+**^ under spectrochemical conditions, finding that this system is 102 ± 8.4% selective for H_2_O_2_ (*n*_cat_ = 2; Figure S22), in contrast to the electrochemical studies. Additional control testing showed no degradation of H_2_O_2_ by disproportionation or catalytic H_2_O_2_ reduction with Cp*_2_Fe as the reductant (Figures S23 & S24). Therefore, based on the apparent rate law, the slope of variable [**im**^**+**^] studies (Figure S18) could be used to estimate an apparent TOF of 6.66 × 10^3^ s^–1^.
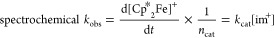
2

**Figure 3:**
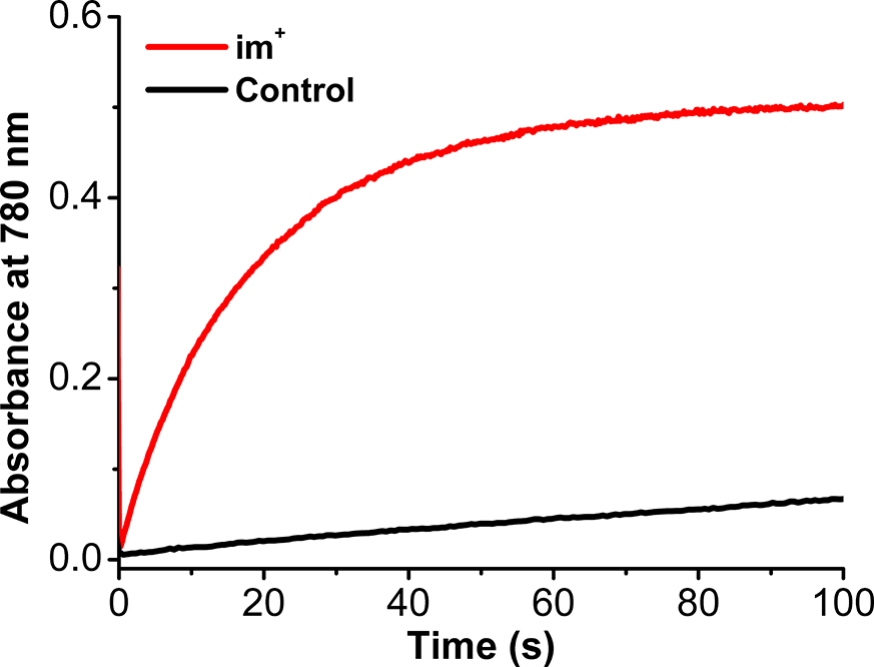
Formation of [Cp*_2_Fe]^+^ at 780 nm from the ORR catalyzed by **im**^**+**^ (red trace) and control (black trace). Concentrations: **im**^**+**^ = 4 μM, TFAH = 25 mM, O_2_ = 4.05 mM, Cp*_2_Fe = 1 mM; control: no **im**^**+**^.

To evaluate the thermodynamics of the reaction, computational studies of likely intermediates during O_2_ reduction were undertaken (see Supporting Information). Evaluation of spin density showed that the radical character of **im**^**0**^ is localized on the imine C atom with some electron density shared by the N atom (Figure S25). Positioning the neutral radical and O_2_ within a sufficient radius for a covalent interaction did not result in bond formation; however, electron transfer between the two occurred resulting in the formation of superoxide O_2_^•–^ and **im**^**+**^, although free energy of the reaction was endergonic overall (+8.8 kcal/mol). In the presence of TFAH (and considering the exergonicity of homoconjugation between TFAH and trifluoroacetate), the formation of protonated superoxide HO_2_^•^ is favorable by −33.2 kcal/mol. Subsequent disproportionation of two equiv of HO_2_^•^ to form O_2_ and H_2_O_2_ is comparably favorable at −33.1 kcal/mol. The reduction potential of HO_2_^•^ is estimated to be 0.58 V negative of the **im**^**+/0**^ couple, excluding outer-sphere reduction.

Since the minimal electrocatalytic current observed with H_2_O_2_ in comparison to that with O_2_ and **im**^**+**^ was not observed to reduce H_2_O_2_ under spectrochemical conditions (Figure S24), subsequent calculations focused on alternative pathways to produce water. The reaction between 1 equiv of **im**^**0**^ and O_2_^•–^ to produce a monoanionic C-bound end-on peroxide species is exergonic by −21.7 kcal/mol. Protonation by TFAH to generate a neutral hydroperoxide is further downhill by −42.0 kcal/mol, considering homoconjugation. The alternative generation of this intermediate by the reaction of **im**^**0**^ and HO_2_^•^ is favorable by −30.6 kcal/mol.

Protonation of the hydroperoxide to generate an oxaziridinium with water coproduct is downhill by an additional −18.2 kcal/mol. The reduction potential of the oxaziridinium is calculated to be approximately 0.21 V more negative than **im**^**+/0**^; however, subsequent protonation to produce a cationic C–OH is favorable by −49.0 kcal/mol. Given the rate of catalysis observed electrochemically, it is probable that these steps occur as a single-proton-coupled electron transfer step, which would be favored overall by −44.2 kcal/mol (+1.92 V vs Fc^+^/Fc). Reduction of this cationic species is expected to be facile, with a calculated reduction potential of +1.34 V versus Fc^+/0^ (Figure S26). The protonation of the resultant neutral C–OH group to generate water and reform **im**^**+**^ is then favorable, with an estimated free energy change of −10.4 kcal/mol.

Based on electrochemical, spectrochemical, and computation analyses, separate cycles for the reduction of dioxygen by **im**^**+**^ under electrochemical and spectrochemical conditions can be proposed (Scheme 1). Starting at *i*, an electron transfer to form carbon-centered radical species, *ii*, which reacts with O_2_ to reform *i* and an equivalent of O_2_^•–^. The product O_2_^•–^ is then protonated by TFAH to form two equivalents of HO_2_^•^, which favorably disproportionate to one equiv each of O_2_ and H_2_O_2_.^20^ Under spectroscopic conditions, the catalytic cycle closes here, as supported by the observed quantitative selectivity for H_2_O_2_ (Figures S22 & S27). Under electrochemical conditions, control experiments show that reactivity with H_2_O_2_ is slow relative to that with O_2_, suggesting that these conditions have a divergent mechanistic pathway. Instead, it is proposed that under electrochemical conditions, the neutral radical *ii* is available in sufficient concentrations in the reaction-diffusion layer to bind available O_2_^•–^, which is supported by the RSD pathway observed in electrochemical studies (Figures S3 & S4). Based on these data and the empirically determined rate law, it is likely that species *iv* represents the resting state of the catalytic cycle, with the protonation reaction to generate *v* representing the rate-determining step (Figure S28).

**Scheme 1:**
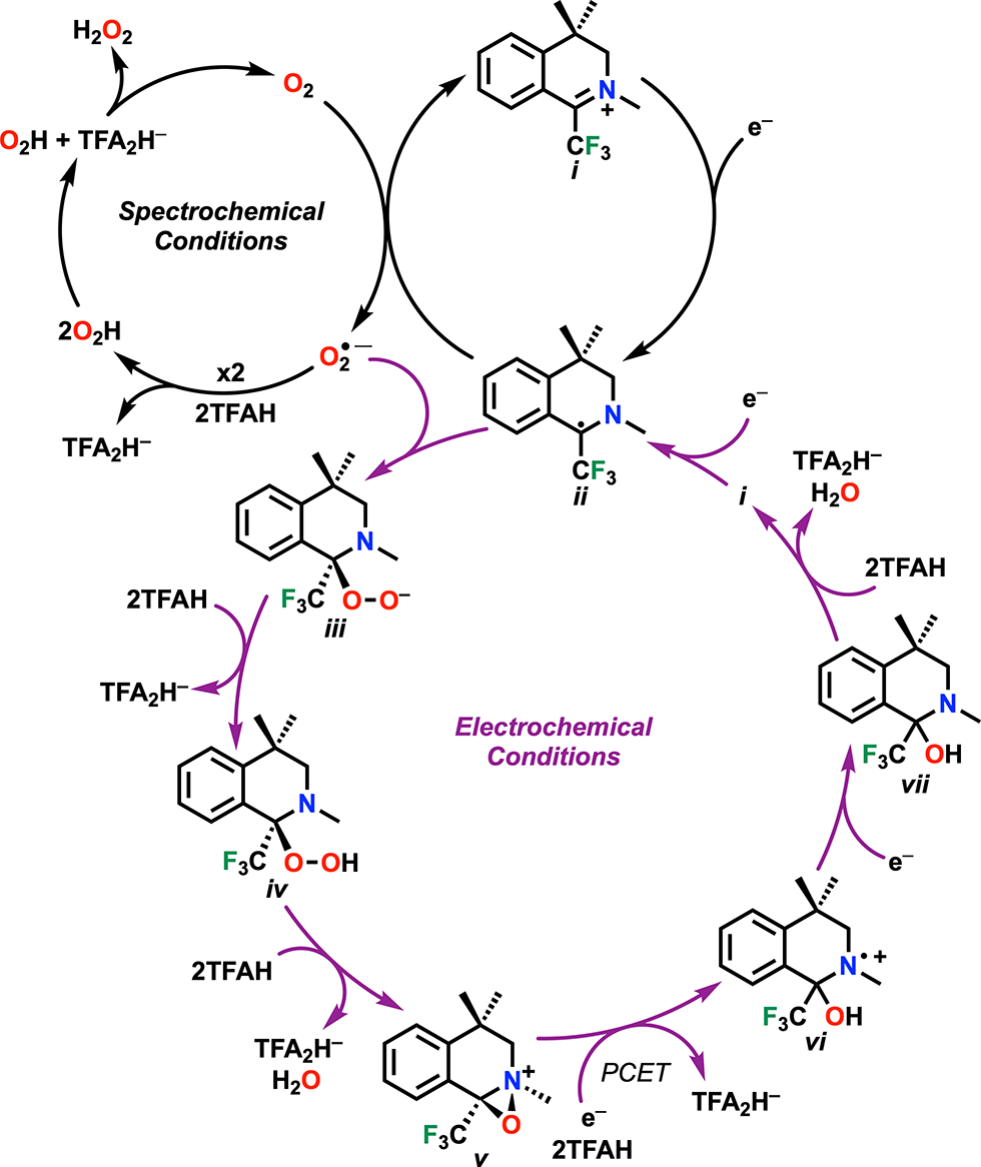
Proposed Catalytic Cycle for ORR by im^+^

The change in accessible pathways can then be ascribed to the concentration differences under each reaction condition. The reaction-diffusion layer during electrochemical experiments is likely to contain both an excess of **im**^**0**^ because of rapid consumption of O_2_ and suitable concentrations of superoxide from rapid outer-sphere reduction. Comparatively, under spectrochemical conditions, the relative concentrations of **im**^**0**^ and O_2_^•–^ are significantly more dilute, allowing thermodynamically viable disproportionation pathways to generate H_2_O_2_. Outer-sphere reduction of HO_2_^•^ by **im**^**0**^ is excluded based on the difference in calculated reduction potentials.

Here, catalytic ORR conditions for H_2_O or H_2_O_2_ production with an iminium-based catalyst have been reported. The difference in accessible reaction pathways under electrochemical and spectrochemical conditions, where the primary product shifts from H_2_O to H_2_O_2_, respectively, is the result of the relative available concentrations of the key **im**^**0**^ and O_2_^•–^ intermediates available under the respective reaction conditions. Since catalysis is initiated by an outer-sphere electron transfer, the O_2_/O_2_^•–^ reduction potential of −1.29 vs Fc^+^/Fc in MeCN defines the overall ORR reaction mediated by **im**^**+**^.^21^ However, the favorable pre-equilibrium reaction between **im**^**0**^ and O_2_^•–^ causes a positive potential shift from this redox couple, indicating that further optimization of the operating potential could be possible.^22^ The work described here reports a novel mechanism whereby the electrocatalytic reduction of O_2_ occurs via both an inner-sphere and outer-sphere mechanism, resulting in product selectivity being controlled by the nature of electron delivery. Given that there are few known organoelectrocatalysts for the ORR,^23^ mechanistic understanding will enable the development of additional examples as well as inform the development of new classes of doped carbons as heterogeneous catalysts.

## Supporting Information Available

Description of experimental details and methods, electrochemical data, and stopped-flow data (PDF)
Computational Coordinates (XYZ)

## Supplementary Material

ja3c14549_si_001.pdf

ja3c14549_si_002.xyz
