# Correction to “Metal-Free Homogeneous O_2_ Reduction by an Iminium-Based Electrocatalyst”

**DOI:** 10.1021/jacs.4c03956

**Published:** 2024-04-16

**Authors:** Emma N. Cook, Anna E. Davis, Michael K. Hilinski, Charles W. Machan

In the *Spectrochemical Conditions* portion of Scheme 1, a duplicated reaction step was incorrectly inserted in the original published version. In the revised version, two equivalents of protonated superoxide, ^•^O_2_H, disproportionate into an equivalent each of H_2_O_2_ and O_2_, as is described in the text. This change does not have any consequence on the original conclusions.

**Scheme 1:**
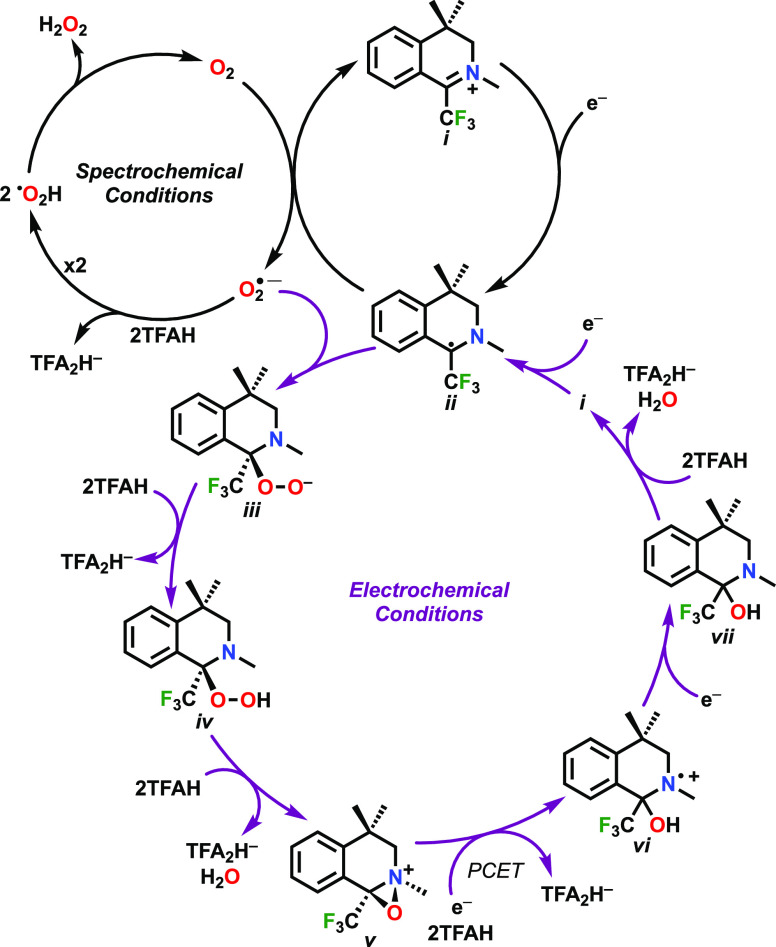
Proposed Catalytic Cycle for ORR by im^+^

