# Supplementary material for: Metal-Free Homogeneous O2 Reduction by an Iminium-Based Electrocatalyst
Source: J Am Chem Soc. 2024 Mar 15;146(12):7931–5. doi: 10.1021/jacs.3c14549 (PMC10979433; doi:10.1021/jacs.3c14549)
Supplement: Supplementary file 1 — ja3c14549_si_001.pdf [file ja3c14549_si_001.pdf]

SUPPORTING INFORMATION for

Metal-Free Homogeneous O_2 Reduction by an Iminium-based Electrocatalyst

Emma N. Cook, Anna E. Davis, Michael K. Hilinski,* Charles W. Machan*

*machan@virginia.edu; ORCID 0000-0002-5182-1138;
*hilinski@virginia.edu; ORCID 0000-0003-2861-7099;

E.N.C ORCID 0000-0002-0568-3600; A.E.D ORCID 0000-0002-6276-8774

Department of Chemistry, University of Virginia,
PO Box 400319, Charlottesville, VA 22904-4319 USA

Table of Contents

General Considerations 4

*Synthesis of Catalyst **im**$^+$* 4

2,2,2-trifluoro-N-(2-methyl-2-phenylpropyl)acetamide (S8) 5

4,4-dimethyl-1-(trifluoromethyl)-3,4-dihydroisoquinoline (S9) 6

3,4-Dihydro-2,4,4-trimethyl-1-(trifluoromethyl)isoquinolinium tetrafluoroborate (im$^+$) . 6

Electrochemical Analysis 6

Figure S1. (A) CVs of **im**$^+$ under Ar saturation at variable scan rates 7

Figure S2. (A) CVs of **im**$^+$ under Ar saturation at varying concentrations. (B) Logarithm of **im**$^+$ concentration versus the reduction peak potential in (A). 8

Figure S3. (A) CVs of **im**$^+$ under O_2 saturation at variable scan rates. (B) Logarithm of scan rate versus reduction peak potential from CVs in (A) 8

Figure S4. (A) CVs of **im**$^+$ under O_2 saturation at varying concentrations. 9

Figure S5. (A) CVs of **im**$^+$ under Ar (black) and O_2 (red) saturation with 0.065 M TFAH added (green). 9

Figure S6. (A) CVs of **im**$^+$ under Ar saturation in the presence of 0.261 M TFAH at variable scan rates. 10

Figure S7. (A) CVs of **im**$^+$ under Ar saturation in the presence of 0.261 M TFAH at varying concentrations. (B) Logarithm of **im**$^+$ concentration versus the reduction peak potential in (A). 10

Figure S8. Rinse test of **im**$^+$ and TFAH 11

Figure S9. CVs of **im**$^+$ under catalytic conditions with variable **im**$^+$ concentrations. 11

Figure S10. CVs of **im**$^+$ under catalytic conditions with variable TFAH concentrations. 12

Figure S11. CVs of **im**$^+$ under catalytic conditions with variable O_2 concentrations 12

Figure S12. CVs of **im**$^+$ with and without added 4.25 mM urea•H_2O_2 under Ar and O_2 saturation. 13

Figure S13. (A) CVs of **im**$^+$ in the presence of 4.25 mM urea•H_2O_2 under Ar and O_2 saturation with added 0.261 M TFAH. (B) CVs of **im**$^+$ in the presence of 4.25 mM urea•H_2O_2 with added 0.261 M TFAH under O_2 saturation. 13

Rotating Ring-Disk Electrode Methods 14

Description of Au Ring Roughening Procedure. 14

Description of RRDE Collection Efficiency. 14

RRDE Experiments 14

Figure S14. Linear sweep voltammograms of RRDE experiment with 0.5 mM im+ and 0.1 M TFAH under Ar (A) and air (B) saturation conditions 15

Figure S15. (A) Levich and Koutecky-Levich (B) plots from data obtained from linear sweep voltammograms of 15

Figure S16. (A) Levich and Koutecky-Levich (B) plots from data obtained from linear sweep voltammograms of 16

Stopped-Flow Spectroscopic Analysis *16*

Figure S17. Change in absorbance at 780 nm over time as a result of the formation of $[Cp^*_2Fe]^+$ by ORR catalyzed by **im$^+$** with TFAH (black trace), example of 1Exp + Mx + C fit in Kinetic Studio 4.0 (red trace), and residual fit (blue trace) 17

Figure S18. Calculated R_{fit}/n_{cat} values from stopped-flow spectrochemical experiments with TFAH, O_2, and Cp^*_2Fe with varying **im$^+$** concentration. 17

Figure S19. Calculated R_{fit}/n_{cat} values from stopped-flow spectrochemical experiments with **im$^+$**, TFAH, Cp^*_2Fe with varying O_2 concentration. 18

Figure S20. Calculated R_{fit}/n_{cat} values from stopped-flow spectrochemical experiments with **im$^+$**, O_2, Cp^*_2Fe with varying TFAH concentration. 18

Figure S21. Calculated R_{fit}/n_{cat} values from stopped-flow spectrochemical experiments with **im$^+$**, TFAH, O_2 with varying Cp^*_2Fe concentration. 19

Spectrochemical Selectivity Determination *19*

Figure S22. H_2O_2 product quantification of ORR by **im$^+$** with TFAH after 2 min 20

Disproportionation Control *20*

Figure S23. Stability test of urea•H_2O_2 in the presence of **im$^+$**, TFAH, and O_2 21

H_2O_2RR Control *21*

Figure S24. Stability test of urea•H_2O_2 in the presence of **im$^+$**, TFAH, and Cp^*_2Fe 22

Computational Methods *22*

Figure S25. (A) spin density plots (0.025 iso) and (B) Kohn-Sham Orbitals (0.05 iso) of the neutral radical **im^0** (S = ½) showing localization at C with contributions from N. Generated from the EPR calculation at the ωB97M-D4/def2-TZVPPD level of theory 23

Figure S26. (A) spin density plots (0.025 iso) and (B) Kohn-Sham Orbitals (0.05 iso) of the neutral radical $C(CF_3)$–OH species (S = ½) showing localization at N. Generated from the EPR calculation at the ωB97M-D4/def2-TZVPPD level of theory 23

Figure S27. Thermodynamics of the catalytic cycle which produces H_2O_2 at an applied potential of –0.87 V vs Fc^+/Fc 24

Figure S28. Thermodynamics of the catalytic cycle which produces H_2O at an applied potential of –0.87 V vs Fc^+/Fc 24

General Considerations

All chemicals and solvents (ACS or HPLC grade) were commercially available and used as received unless otherwise indicated. For all air-sensitive reactions and electrochemical experiments, HPLC-grade solvents were obtained as anhydrous and air-free from a PPT Glass Contour Solvent Purification System. Gas cylinders were obtained from Praxair (Ar as 5.0; O_2 as 4.0) and passed through activated molecular sieves prior to use. Gas mixing for variable concentration experiments was accomplished using a gas proportioning rotameter from Omega Engineering. UV-vis absorbance spectra were obtained on a Cary 60 from Agilent using a quartz cuvette with 1 cm pathlength. The concentration of O_2 saturation in MeCN is reported to be 8.1 mM and the saturation concentration in MeCN with added electrolyte to be 6.3 mM.[1] Flash column chromatography was performed using silica gel or alumina gel (230 - 400 mesh) purchased from Fisher Scientific. Elution of compounds was monitored by UV. 1H and 13C NMR spectra were measured on a Varian Inova 600 (600 MHz) or Bruker Avance III 800 (800 MHz) spectrometer and acquired at 300 K. Chemical shifts are reported in parts per million (ppm δ) referenced to the residual 1H or 13C resonance of the solvent. The following abbreviations are used to indicate the multiplicity of signals: s - singlet, d - doublet, t - triplet, q - quartet, m - multiplet and br - broad.

*Synthesis of **im**$^+$ Catalyst*

Procedures taken and adapted from "Improved Parent Iminium Synthesis Procedure."[2]

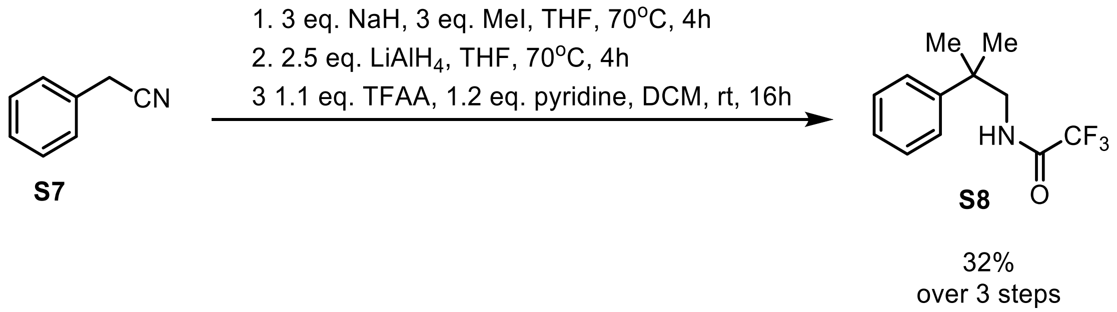

Under N_2 atmosphere, benzyl cyanide (**S7**, 1 equiv) was added into a flame-dried round-bottom flask equipped with a stir bar. Tetrahydrofuran (0.4 M) was added and the suspension was cooled to 0 °C with stirring. 60% sodium hydride (dispersion in paraffin liquid) (3 equiv) was added to the mixture and stirred for 1 hour. Iodomethane (3 equiv) was then added dropwise to the solution at 0°C and the solution was heated to 70 °C for 4 hours. Upon completion, the crude reaction mixture was cooled to room temperature then quenched with ice. The organic layer was extracted 3 times with ethyl acetate, and the combined organics were washed with brine, dried over anhydrous $MgSO_4$, and concentrated *in vacuo*. The crude product was carried on to the next step.

Under N_2 atmosphere, crude methylated benzyl cyanide (1 equiv) was dissolved in tetrahydrofuran (0.4 M) in a round bottom equipped with a stir bar and cooled to 0 °C with stirring. The solution was cooled to 0 °C and 2.4 M solution of lithium aluminum hydride in tetrahydrofuran (2.5 equiv) was added dropwise by addition funnel. The solution was heated to 70 °C and stirred for 4 hours. Upon completion, the crude reaction mixture was worked up following the Fieser Method: The white suspension was cooled to 0°C and diluted to roughly twice its volume with diethyl ether. "x" mL water was slowly added to the reaction mixture, where "x" is the amount of lithium aluminum hydride used for the reduction in grams. "x" mL 15% aqueous sodium hydroxide

was then added, followed by “3x” mL water. The mixture was then warmed to rt and stirred 15 minutes, followed by addition of anhydrous $MgSO_4$. Upon stirring for an additional 15 minutes, the mixture was filtered over a pad of celite and concentrated *in vacuo*. The crude product was carried onto the next step.

Under N_2 atmosphere, the crude amine (1 equiv) was dissolved in anhydrous dichloromethane (0.1 M) in a round-bottom flask equipped with a stir bar. Pyridine (1.2 equiv) was added with stirring, followed by dropwise addition of trifluoroacetic anhydride (1.1 equiv) via syringe. The reaction mixture was stirred at room temperature overnight (ca. 16 h). Upon completion, the orange-brown solution was quenched with a brine wash, dried over $MgSO_4$, and concentrated *in vacuo*. The residue was purified by flash chromatography with isocratic 20% ethyl acetate in hexanes to give acetamide **S8** a white crystalline solid (32% yield over 3 steps).

2,2,2-trifluoro-N-(2-methyl-2-phenylpropyl)acetamide (S8)

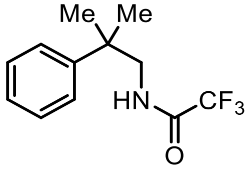

1H NMR (600 MHz, $CDCl_3$): δ 7.33 – 7.38 (m, 3H), 7.25 – 7.27 (m, 1H), 5.89 (br. s, 1H), 3.53 (d, J = 4.8 Hz, 2H), 1.37 (s, 6H).

13C NMR (151 MHz, $CDCl_3$): δ 157.1, 145.0, 128.9, 126.8, 125.7, 50.9, 38.7, 26.3.

NMR spectra are consistent with literature reports.[3]

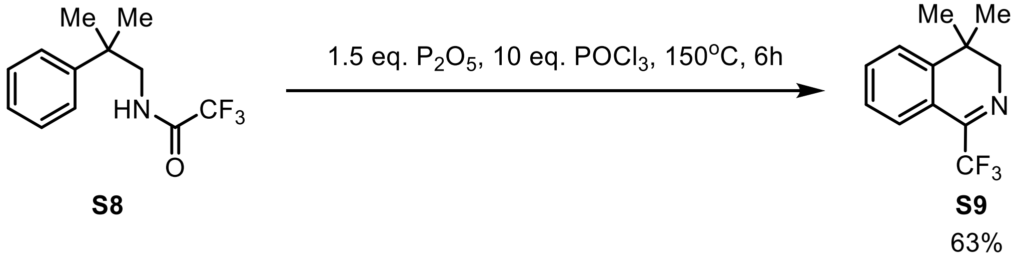

Under N_2 atmosphere, phosphorus pentoxide (1.5 equiv) was added to a 2-neck round-bottom flask equipped with a stir bar. Half the volume of phosphorus oxychloride (5 equiv) was added to the solution and heated to 70 °C, then immediately heated to 120 °C. The acetamide (**S8**, 1 equiv) was dissolved in the remaining half volume (5 equiv) of phosphorus oxychloride and added to the solution slowly. The solution turned brown and then black, and was further heated to 150 °C and allowed to stir for 5 hours. Upon completion, the mixture was cooled to room temperature, carefully diluted to twice the original volume with dichloromethane, and transferred to a large Erlenmeyer flask. In the flask, the brown-black solution was quenched slowly with excess water, then saturated aqueous sodium bicarbonate with stirring. The mixture was treated with base until it was light tan in color and the pH measured at 8 or greater. The resultant solution was extracted 3 times with dichloromethane and the combined organic layers were washed with brine, dried over $MgSO_4$, and concentrated *in vacuo*. The crude residue was purified by flash chromatography with 0 – 5% ethyl acetate/hexanes to give the cyclized imine as a yellow oil (**S9**, 63% yield).

4,4-dimethyl-1-(trifluoromethyl)-3,4-dihydroisoquinoline (S9)

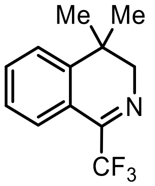

1H NMR (600 MHz, $CDCl_3$): δ 7.60 – 7.63 (m, 1H), 7.49 – 7.51 (m, 1H), 7.40 – 7.42 (m, 1H), 7.31 – 7.34 (m, 1H), 3.74 (d, J = 4.8 Hz, 2H), 1.24 (s, 6H).

13C NMR (151 MHz, $CDCl_3$): δ 155.6, 146.7, 132.7, 126.7, 125.7, 124.0, 121.1, 59.9, 31.7, 26.0.

NMR spectra are consistent with literature reports.[3]

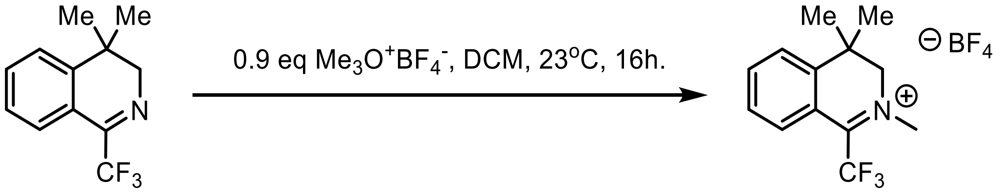

In an N_2 glovebox, imine (**S9**, 1 equiv) was dissolved in anhydrous dichloromethane (0.5 M) in a vial equipped with a stir bar. Trimethyloxonium tetrafluoroborate (0.9 equiv) was added to the solution and stirred at room temperature overnight (ca.16 h) and removed from the glovebox. Solvent was removed *in vacuo* and the resulting solid was washed with anhydrous diethyl ether, then recrystallized from dichloromethane and diethyl ether to yield the iminium catalyst as a white crystalline solid (**im**$^+$, 90% yield).

3,4-Dihydro-2,4,4-trimethyl-1-(trifluoromethyl)isoquinolinium tetrafluoroborate (im$^+$)

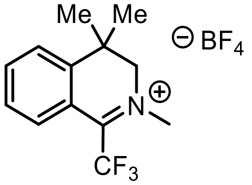

1H NMR (600 MHz, $CDCl_3$): δ 8.03 – 8.11 (m, 2H), 8.03 – 8.04 (m, 1H), 7.69 – 7.85 (m, 1H), 4.45 (s, 2H), 4.30 (s, 3H), 1.49 (s, 6H).

13C NMR (151 MHz, $CDCl_3$): δ 148.0, 139.7, 131.2, 128.3, 125.2, 121.4, 119.3, 65.8, 48.9, 33.0, 24.1.

NMR spectra are consistent with literature reports.[3]

Electrochemical Analysis

All cyclic voltammetry experiments were performed using a Metrohm Autolab PGSTAT302N potentiostat. Glassy carbon working (⌀ = 3 mm) and non-aqueous silver/silver chloride pseudoreference electrodes behind PTFE tips were obtained from CH Instruments. The pseudoreference electrodes were obtained by depositing chloride on bare silver wire in 10% HCl

at oxidizing potentials and stored in a 0.1 M tetrabutylammonium hexafluorophosphate solution in acetonitrile in the dark prior to use. The counter electrode was a glassy carbon rod (⌀ = 3 mm). All CV experiments were performed in a modified scintillation vial (20 mL volume) as a single-chamber cell with a cap modified with ports for all electrodes and a sparging needle. Tetrabutylammonium hexafluorophosphate ($TBAPF_6$) was purified by recrystallization from ethanol and dried in a vacuum oven before being stored in a desiccator. All data were referenced to an internal ferrocene standard (ferrocenium/ferrocene reduction potential under stated conditions) unless otherwise specified. All voltammograms were corrected for internal resistance. Ferrocene was purified by sublimation prior to use. In the event that the presence of electrochemical features precluded ferrocene addition, ferrocene was added to the electrochemical cell at the end of analysis for reference. All CVs were scanned to negative potentials before sweeping to positive potentials. Rotating ring-disk electrode electroanalytical experiments were performed using a BioLogic VSP Bipotentiostat and a Pine Research MSR Rotator. Glassy carbon working electrode (⌀ = 5 mm) with a gold ring were obtained from Pine Research.

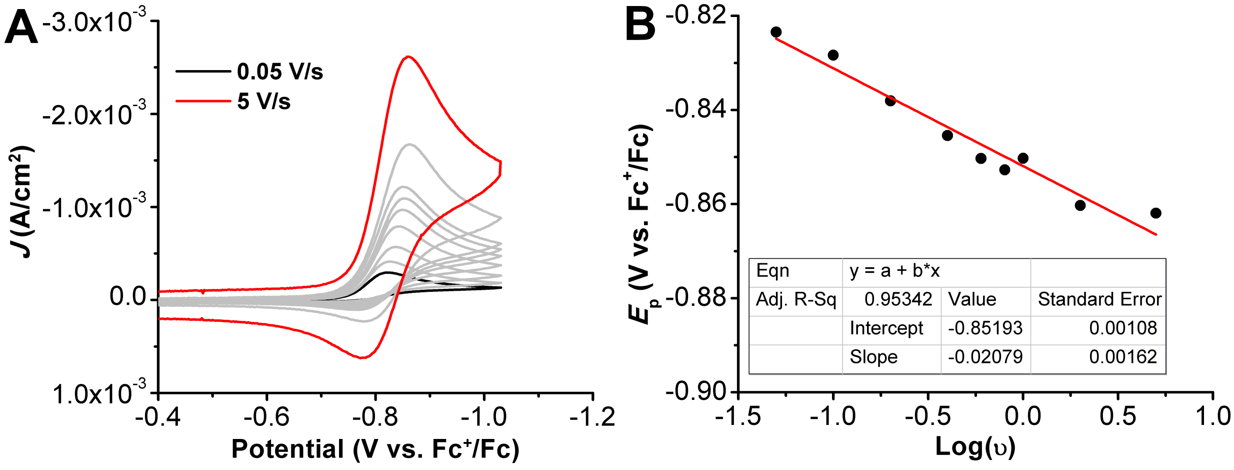

Figure S1. (A) CVs of **im⁺** under Ar saturation at variable scan rates. (B) Logarithm of scan rate versus reduction peak potential from CVs in (A). Conditions: 1.3 mM **im⁺**, 0.1 M $TBAPF_6$/MeCN; glassy carbon working electrode, glassy carbon counter electrode, Ag/AgCl pseudoreference electrode; referenced to an internal ferrocene standard. Scan rates: 0.05, 0.1, 0.2, 0.4, 0.6, 0.8 1, 2, 5 V/s.

Mechanism	λ	$\frac{\delta E_p}{\delta \log v}$ (*)	$\frac{\delta E_p}{\delta \log C^0}$ (*)	$E_p - E^0$
EC	$\frac{RT}{F}\frac{k_+}{\upsilon}$	–29.6	0.0	$-0.780\frac{RT}{F}+\frac{RT}{2F}ln\left(\frac{RT}{F}\frac{k_+}{\upsilon}\right)$
RRD	$\frac{RT}{F}\frac{k_d C^0}{\upsilon}$	–19.7	19.7	$-0.903\frac{RT}{F}+\frac{RT}{3F}ln\left(\frac{4RT}{3F}\frac{k_d C^0}{\upsilon}\right)$

(*) – at 25 °C.[4] For **Figures S1 and S2**.

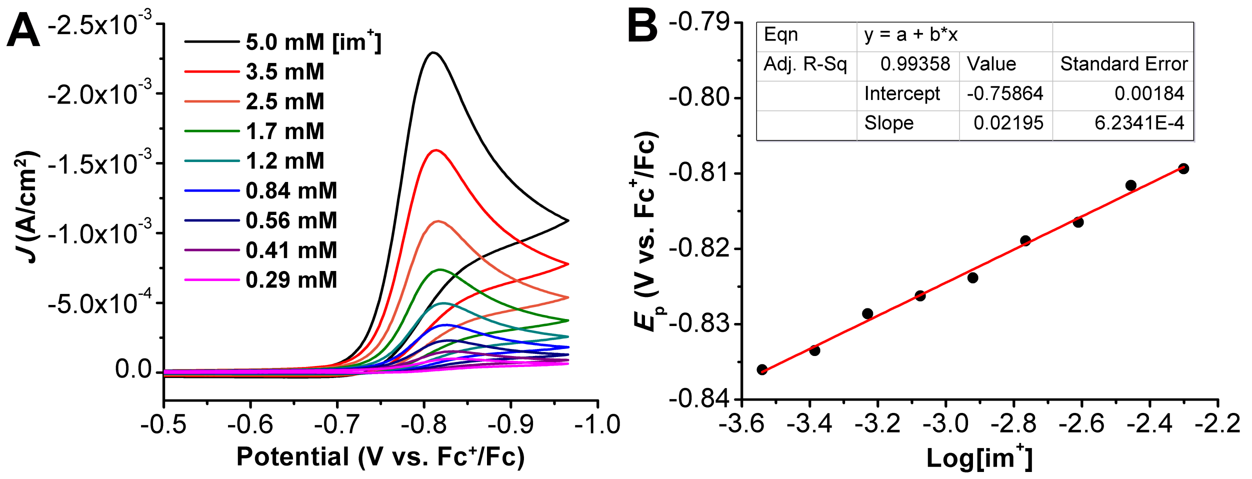

Figure S2. (A) CVs of **im**$^+$ under Ar saturation at varying concentrations. (B) Logarithm of **im**$^+$ concentration versus the reduction peak potential in (A). Conditions: varying [im$^+$], 0.1 M $TBAPF_6$/MeCN; glassy carbon working electrode, glassy carbon counter electrode, Ag/AgCl pseudoreference electrode; referenced to an internal ferrocene standard.

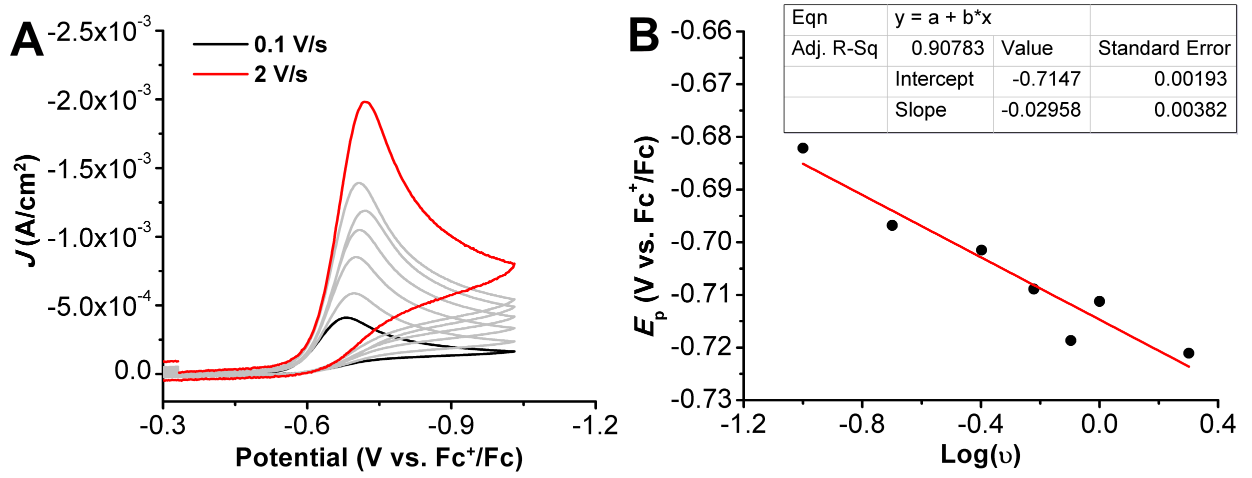

Figure S3. (A) CVs of **im**$^+$ under O_2 saturation at variable scan rates. (B) Logarithm of scan rate versus reduction peak potential from CVs in (A). Conditions: 1.3 mM **im**$^+$, 0.1 M $TBAPF_6$/MeCN; glassy carbon working electrode, glassy carbon counter electrode, Ag/AgCl pseudoreference electrode; referenced to an internal ferrocene standard. Scan rates: 0.1, 0.2, 0.4, 0.6, 0.8 1, 2 V/s.

Mechanism	λ	$\frac{\delta E_p}{\delta \log \nu}$ (*)	$\frac{\delta E_p}{\delta \log C^0}$ (*)	$E_p - E^0$
RSD-ECE	$\frac{RT}{F}\frac{k_d C^0}{\upsilon}$	–29.6	29.6	$-1.15\frac{RT}{F} + \frac{RT}{2F} ln\left(\frac{4RT}{F}\frac{k_d C^0}{\upsilon}\right)$
RSD-DISP1	$\frac{RT}{F}\frac{k_d C^0}{\upsilon}$	–29.6	29.6	$-1.15\frac{RT}{F} + \frac{RT}{2F} ln\left(\frac{2RT}{F}\frac{k_d C^0}{\upsilon}\right)$

(*) – at 25 ºC.[4] For **Figures S3-S7**.

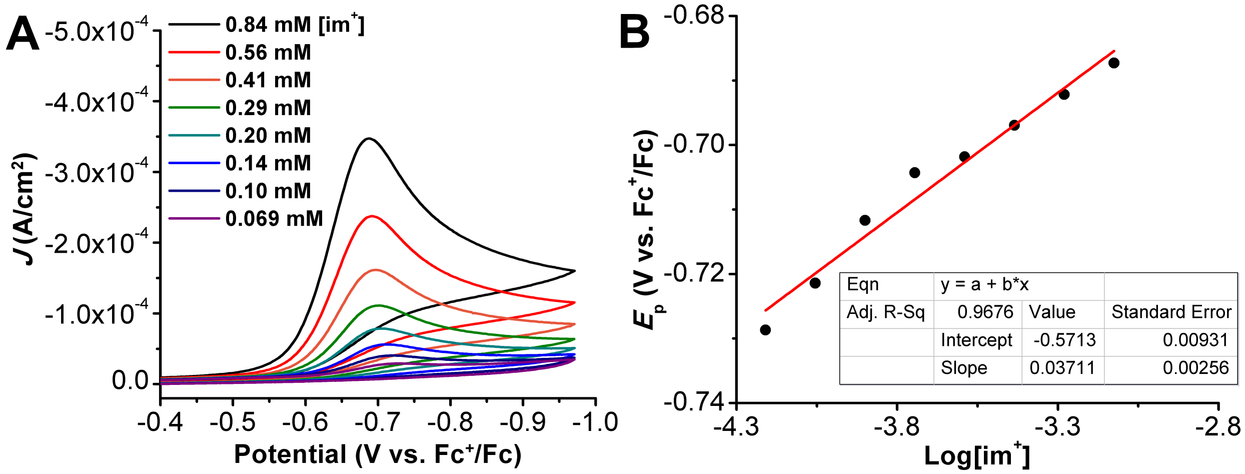

Figure S4. (A) CVs of **im$^+$** under O_2 saturation at varying concentrations. (B) Logarithm of im+ concentration versus the reduction peak potential in (A). Conditions: varying [im+], 0.1 M TBAPF6/MeCN; glassy carbon working electrode, glassy carbon counter electrode, Ag/AgCl pseudoreference electrode; referenced to an internal ferrocene standard.

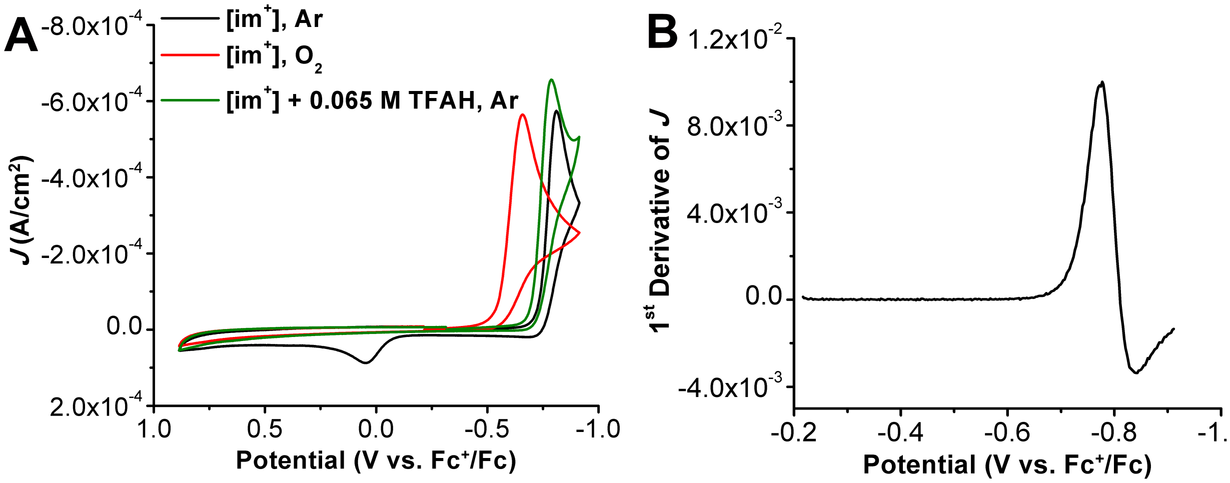

Figure S5. (A) CVs of **im$^+$** under Ar (black) and O_2 (red) saturation with 0.065 M TFAH added (green). (B) First derivative of current density of **im$^+$** under Ar saturation (from black trace in A) Conditions: Conditions: 1.3 mM **im$^+$**, 0.1 M $TBAPF_6$/MeCN; 100 mV/s; glassy carbon working electrode, glassy carbon counter electrode, Ag/AgCl pseudoreference electrode; referenced to an internal ferrocene standard.

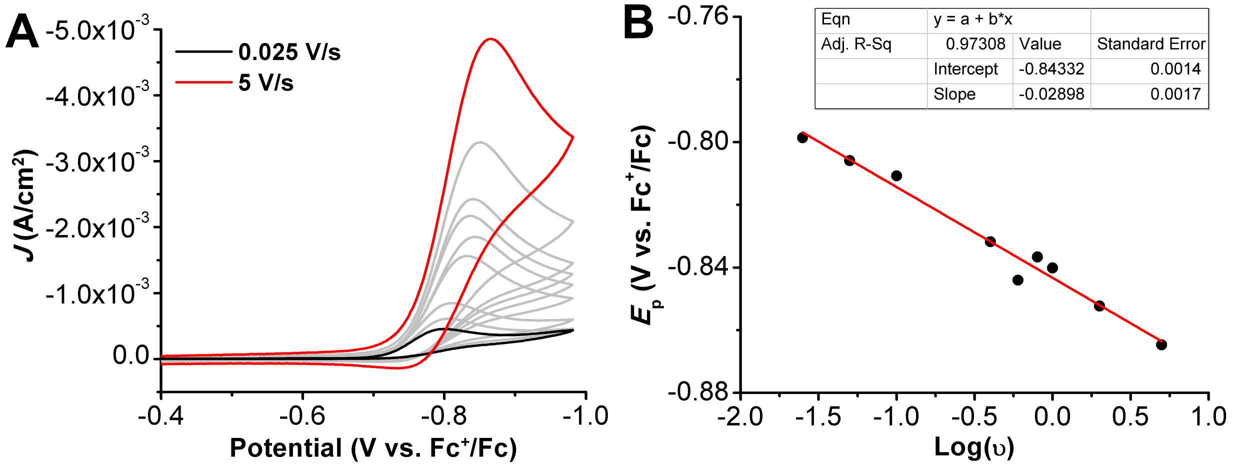

Figure S6. (A) CVs of **im**$^{+}$ under Ar saturation in the presence of 0.261 M TFAH at variable scan rates. (B) Logarithm of scan rate versus reduction peak potential from CVs in (A). Conditions: 1.3 mM **im**$^{+}$, 0.261 M TFAH, 0.1 M $TBAPF_6$/MeCN; glassy carbon working electrode, glassy carbon counter electrode, Ag/AgCl pseudoreference electrode; referenced to an internal ferrocene standard. Scan rates: 0.025, 0.05, 0.1, 0.4, 0.6, 0.8 1, 2, 5 V/s.

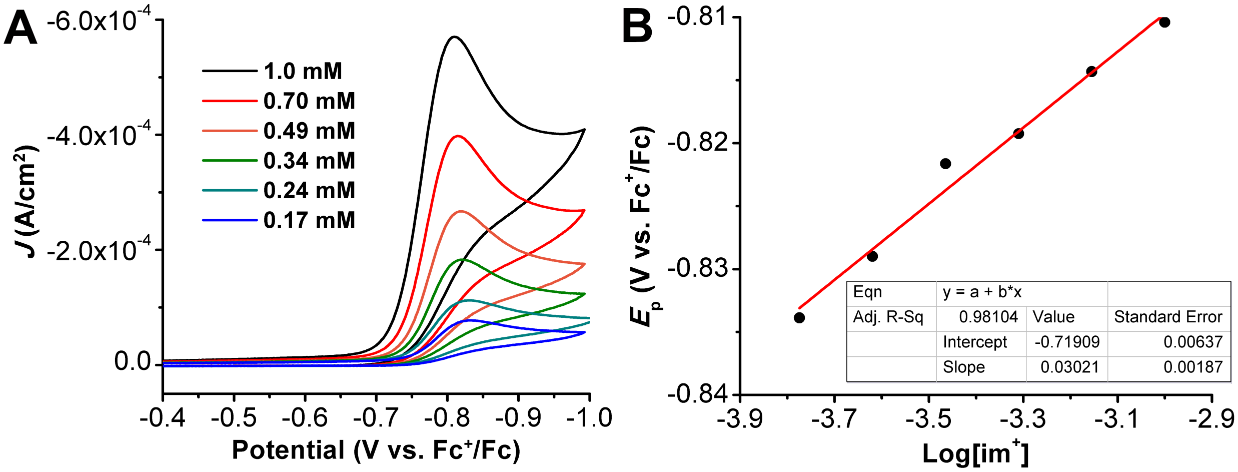

Figure S7. (A) CVs of **im**$^{+}$ under Ar saturation in the presence of 0.261 M TFAH at varying concentrations. (B) Logarithm of **im**$^{+}$ concentration versus the reduction peak potential in (A). Conditions: varying [**im**$^{+}$], 0.261 M TFAH, 0.1 M $TBAPF_6$/MeCN; glassy carbon working electrode, glassy carbon counter electrode, Ag/AgCl pseudoreference electrode; referenced to an internal ferrocene standard.

To ensure that species adsorbed to the electrode, a rinse test was performed with **im$^+$** and TFAH (**Figure S8**). A CV was taken under catalytic conditions after which the electrode was removed and the sides were wiped and placed in a blank solution containing TFAH and a CV was taken.

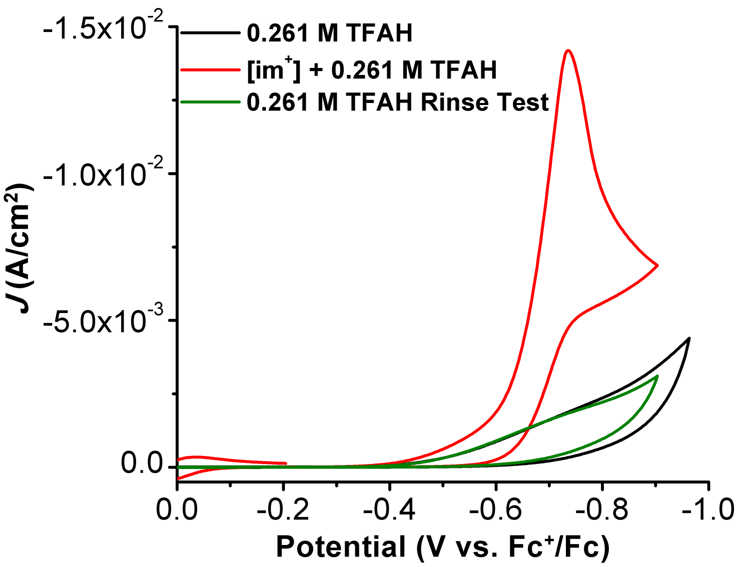

Figure S8. Rinse test of **im$^+$** and TFAH. CV of TFAH under O_2 saturation (black trace), **im$^+$** under catalytic conditions (red trace), and rinse test (green trace). Conditions: 1.3 mM **im$^+$** 0.261 M TFAH, 0.1 M $TBAPF_6$/MeCN, O_2 saturation; 100 mV/s; glassy carbon working electrode, glassy carbon counter electrode, Ag/AgCl pseudoreference electrode; referenced to an internal ferrocene standard.

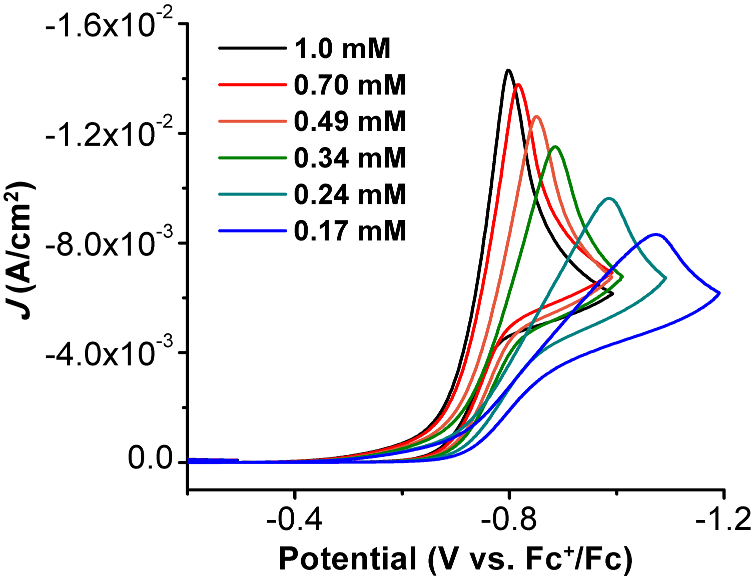

Figure S9. CVs of **im$^+$** under catalytic conditions with variable **im$^+$** concentrations. Conditions: 0.261 M TFAH, 0.1 M $TBAPF_6$/MeCN; 100 mV/s; glassy carbon working electrode, glassy carbon counter electrode, Ag/AgCl pseudoreference electrode; referenced to an internal ferrocene standard.

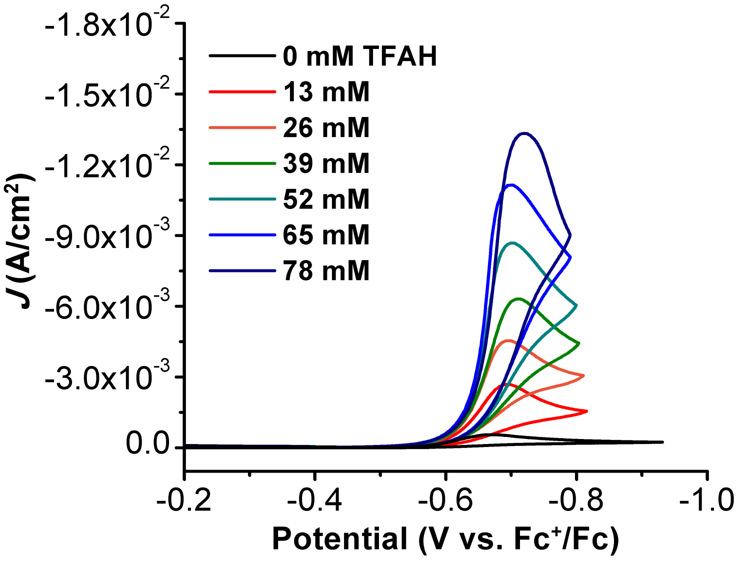

Figure S10. CVs of **im⁺** under catalytic conditions with variable TFAH concentrations. Conditions: 1.3 mM **im⁺**, 0.1 M $TBAPF_6$/MeCN; 100 mV/s; glassy carbon working electrode, glassy carbon counter electrode, Ag/AgCl pseudoreference electrode; referenced to an internal ferrocene standard.

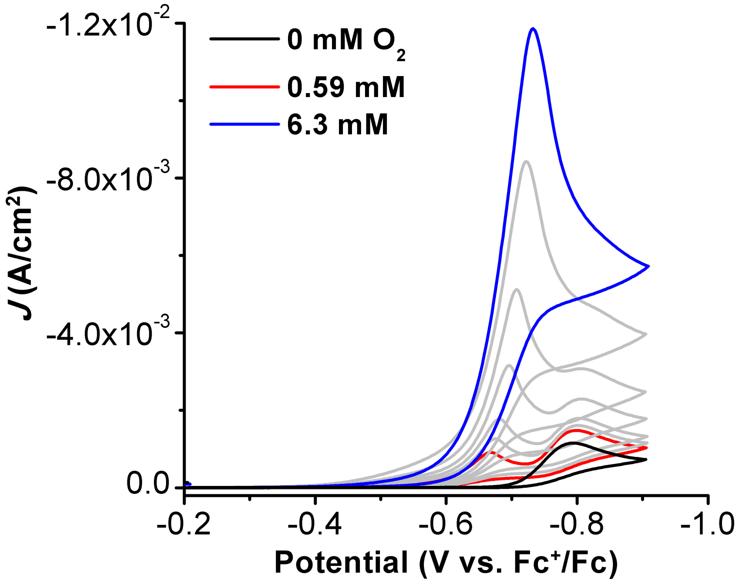

Figure S11. CVs of **im⁺** under catalytic conditions with variable O_2 concentrations. Conditions: 1.3 mM **im⁺**, 0.261 M TFAH, 0.1 M $TBAPF_6$/MeCN; 100 mV/s; glassy carbon working electrode, glassy carbon counter electrode, Ag/AgCl pseudoreference electrode; referenced to an internal ferrocene standard. O_2 concentrations: 6.3, 5.06, 3.3, 1.45, 0.92, 0.74, 0.59 mM.

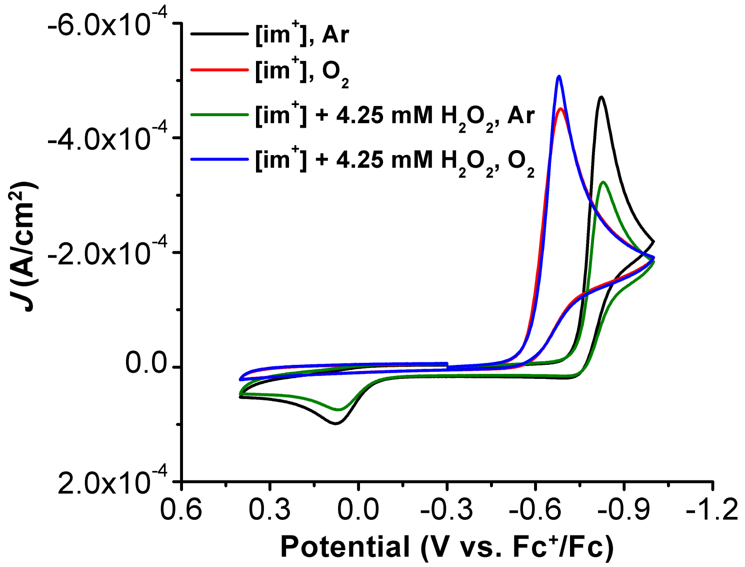

Figure S12. CVs of **im⁺** with and without added 4.25 mM urea•H_2O_2 under Ar and O_2 saturation. Conditions: 1.3 mM **im⁺**, 0.261 M TFAH, 0.1 M $TBAPF_6$/MeCN; 100 mV/s; glassy carbon working electrode, glassy carbon counter electrode, Ag/AgCl pseudoreference electrode; referenced to an internal ferrocene standard.

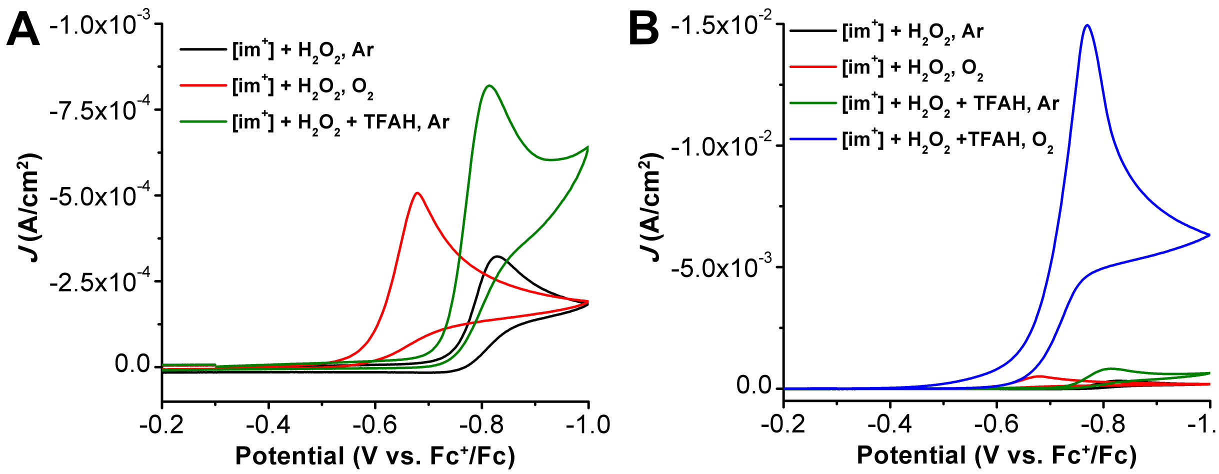

Figure S13. (A) CVs of **im⁺** in the presence of 4.25 mM urea•H_2O_2 under Ar and O_2 saturation with added 0.261 M TFAH. (B) CVs of **im⁺** in the presence of 4.25 mM urea•H_2O_2 with added 0.261 M TFAH under O_2 saturation. Conditions: 1.3 mM **im⁺**, 0.261 M TFAH, 0.1 M $TBAPF_6$/MeCN; 100 mV/s; glassy carbon working electrode, glassy carbon counter electrode, Ag/AgCl pseudoreference electrode; referenced to an internal ferrocene standard.

Rotating Ring-Disk Electrode Methods

Description of Au Ring Roughening Procedure. The Au ring electrode was roughened according to a previously reported method.[5] The electrodes were polished first on a felt polishing pad with 0.3 micron alumina, then with 0.05 micron alumina and rinsed with water and ethanol. Cyclic voltammograms were obtained in 0.5 M H_2SO_4 by scanning from 0 to 1.6 V vs. Ag/AgCl at 100 mV/s, then at 20 mV/s for an additional 2 cycles to obtain the pre-roughening, surface oxide reduction charge. The electrode was then pulsed between 2.4 and 0.2 V vs Ag/AgCl for 2.4 ms each and repeated for 250,000 cycles. Bubbles formed during electrolysis pulses were dislodged by contacting with a large bubble from a glass pipette. After electrolysis, the electrode was held at 0.3 V vs. Ag/AgCl for 2 minutes and the roughening was evaluated by CV.

Description of RRDE Collection Efficiency. The collection efficiency was determined as previously reported.[6-8] Conditions: Ar saturation, 0.1 M $TBAPF_6$, 0.5 mM ferrocene in MeCN, glassy carbon disk electrode (5 mm), roughened Au ring electrode, glassy carbon rod counter electrode, Ag/AgCl pseudoreference electrode; scan rate 0.1 V/s. To calculate the collection efficiency of the RRDE, the ratio of the ring current (i_r) to the disk current (i_d) at each rotation rate was used to determine $N_{empirical}$ (**Eq S1**). The $N_{empirical}$ value at each rotation rate was multiplied by a factor of 100 to determine the collection efficiency % at each rotation rate (~15%).

$$N_{empirical} = \frac{i_{ring\ corrected}}{i_{disk\ corrected}} \qquad \textbf{(Eq S1)}$$

RRDE Experiments. Conditions: Performed under Ar and air saturation conditions, 0.1 M $TBAPF_6$, 0.5 mM **im$^+$**, 0.1 M TFAH, glassy carbon disk electrode (5 mm diameter), roughened Au ring electrode, glassy carbon rod counter electrode, Ag/AgCl pseudoreference electrode; scan rate 0.1 V/s.

The solution was sparged until saturation was achieved. **Im$^+$** (0.5 mM) was dissolved in solution and 0.1 M TFAH was added. A standard CV was taken of the solution to confirm the potential window to be used for the experiment. The roughened Au ring was set to +1.2 V. LSVs were obtained for various rotation rates between 400 and 2400 under the described conditions. In between each scan, the solution was sparged for 3 minutes. The reproducibility of scans was confirmed by repeating scans at the same rotation rate, producing exact overlays. The same procedure was repeated for air saturation conditions, which were achieved by sparging the solution with air for 3 minutes. Disk (i_d) and ring (i_r) currents were corrected by subtracting the current observed under Ar to ensure that the current observed was a result of H_2O_2 formation.

The arithmetic mean of the number of electrons received by O_2 (n_{cat}) during the ORR was calculated from the disk current (i_d) and ring current (i_r) according to **Eq S2:**

$$n_{cat} = 4 \times \frac{i_d}{i_d + \frac{i_r}{N_{empirical}}} \qquad \textbf{(Eq S2)}$$

The H_2O_2 ratio (p) is defined as the fraction of O_2 reduced to H_2O_2 and relates to n_{cat} by **Eq S3**:

$$n_{cat} = 4 - 2p \qquad \textbf{(Eq S3)}$$

Multiplying p by 100% provides the %H_2O_2 selectivity of the ORR. It was determined that under electrochemical conditions, this system shows a 7.50 ± 1.3% selectivity for H_2O_2.

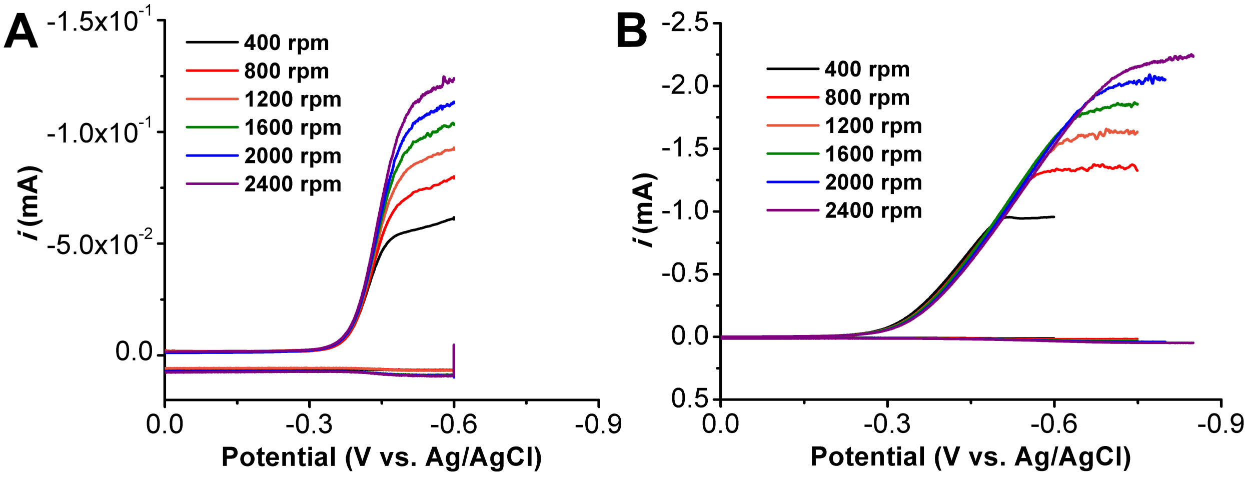

Figure S14. Linear sweep voltammograms of RRDE experiment with 0.5 mM im+ and 0.1 M TFAH under Ar (A) and air (B) saturation conditions; ring potential = 1.2 V vs Ag/AgCl. Conditions: 0.5 mM **im$^+$**, 0.1 M TFAH, 0.1 M $TBAPF_6$/MeCN; glassy carbon working electrode/roughened Au ring working electrode, glassy carbon counter electrode, Ag/AgCl pseudoreference electrode; scan rate 0.1 V/s.

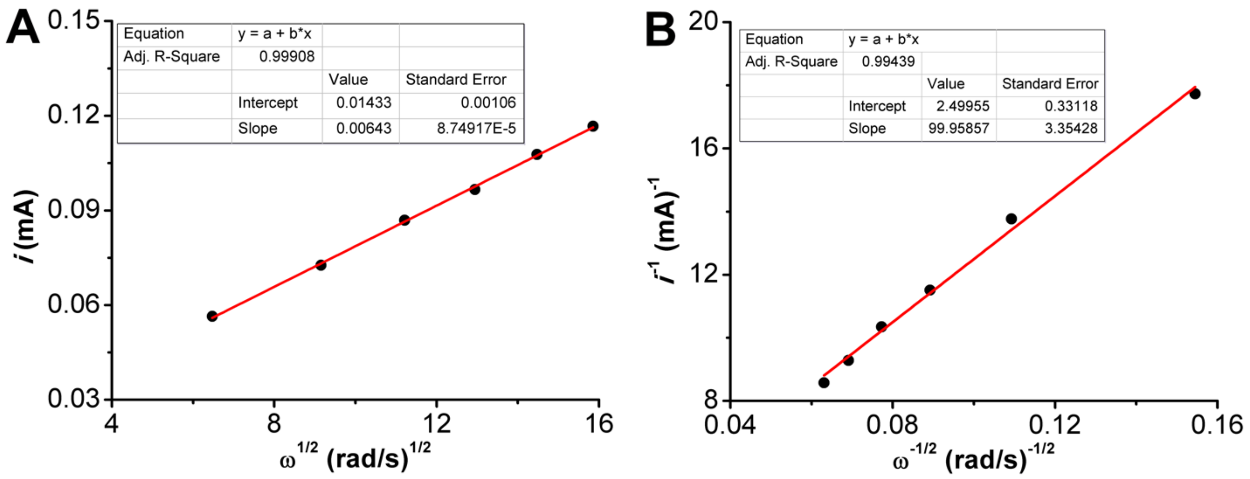

Figure S15. (A) Levich and Koutecky-Levich (B) plots from data obtained from linear sweep voltammograms of **im$^+$** (0.5 mM) by RRDE with 0.1 M TFAH under Ar saturation conditions at various rotation rates; ring potential = 1.2 V vs. Ag/AgCl.

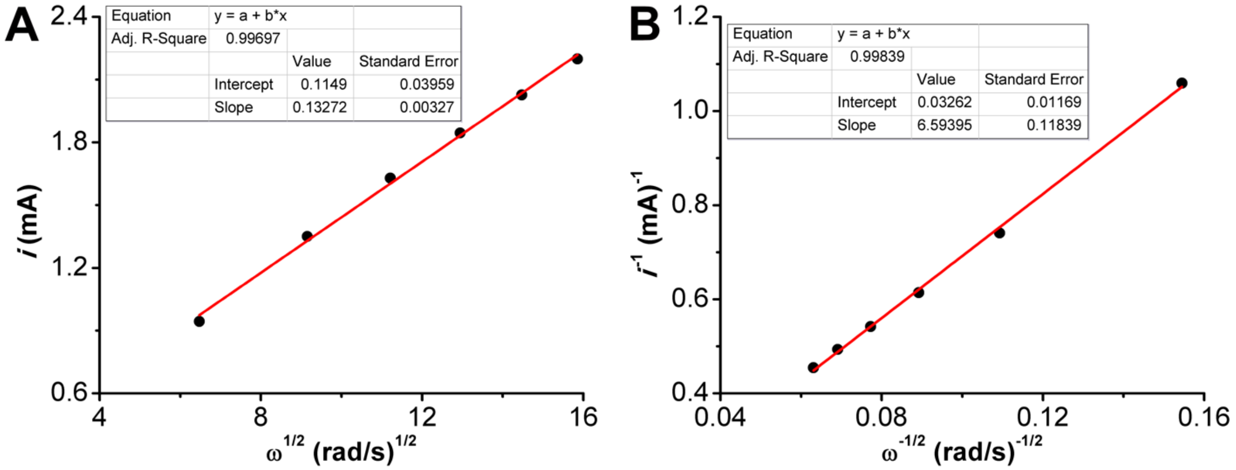

Figure S16. (A) Levich and Koutecky-Levich (B) plots from data obtained from linear sweep voltammograms of **im⁺** (0.5 mM) by RRDE with 0.1 M TFAH under air saturation conditions at various rotation rates; ring potential = 1.2 V vs. Ag/AgCl.

Stopped-Flow Spectroscopic Analysis

Stopped-flow spectrochemical kinetics studies were performed with a CSF-61DX2 Stopped-Flow System from Hi-Tech Scientific. Kinetic Studio Software was used to monitor a single wavelength and Integrated CCD Software was used to monitor the entire visible spectrum. All data fits were performed within the Kinetic Studio 4.0 Software Suite. Prior to experiments, dried and degassed MeCN was passed through syringes and the cell block before reagents were loaded. In a typical experiment, syringes would be charged with known concentrations of reagent. All reagent solutions were prepared immediately before use.

In general, a vial containing **im⁺** catalyst and TFAH was sparged with O_2, drawn into a syringe and loaded into the stopped-flow. A second syringe containing N_2-saturated Cp^*_2Fe solution was loaded into the stopped-flow. All reported concentrations are the mixed concentrations in the spectroscopic cell.

$$\boldsymbol{rate = k_{cat}[im^+]^1[TFAH]^0[O_2]^0[Cp^*{}_2Fe]^0}$$

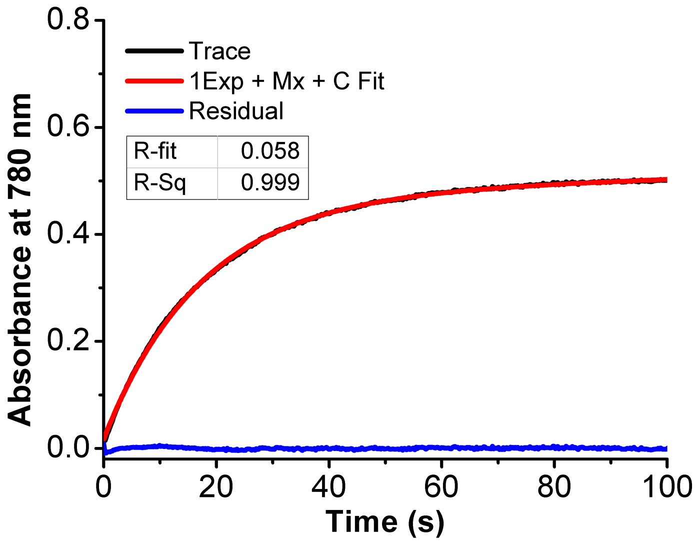

Figure S17. Change in absorbance at 780 nm over time as a result of the formation of $[Cp^*{}_2Fe]^+$ by ORR catalyzed by **im$^+$** with TFAH (black trace), example of 1Exp + Mx + C fit in Kinetic Studio 4.0 (red trace), and residual fit (blue trace). Concentrations: **im$^+$** = 4 μM, TFAH = 25 mM, O_2 = 4.05 mM, $Cp^*{}_2Fe$ = 1 mM.

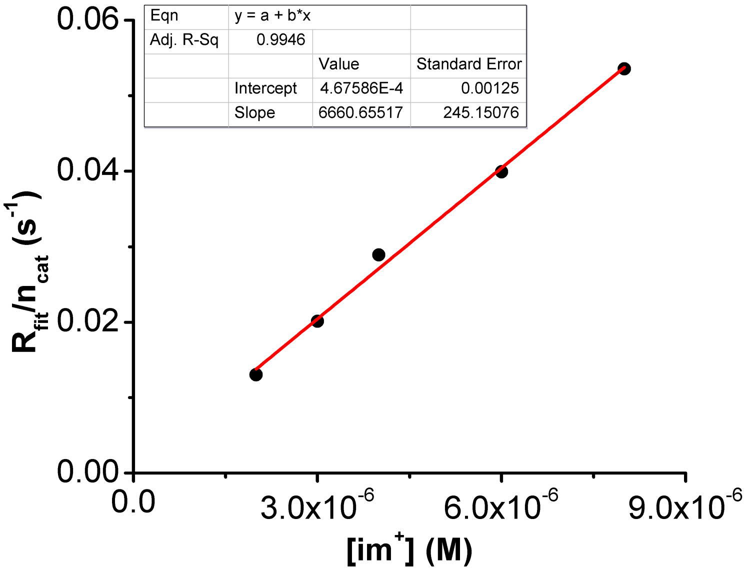

Figure S18. Calculated R_{fit}/n_{cat} values from stopped-flow spectrochemical experiments with TFAH, O_2, and $Cp^*{}_2Fe$ with varying **im$^+$** concentration. Data were fit using Kinetic Studio 4.0 (1Exp+Mx+C); n_{cat} = 2. Concentrations: TFAH = 25 mM, O_2 = 4.05 mM, $Cp^*{}_2Fe$ = 1 mM.

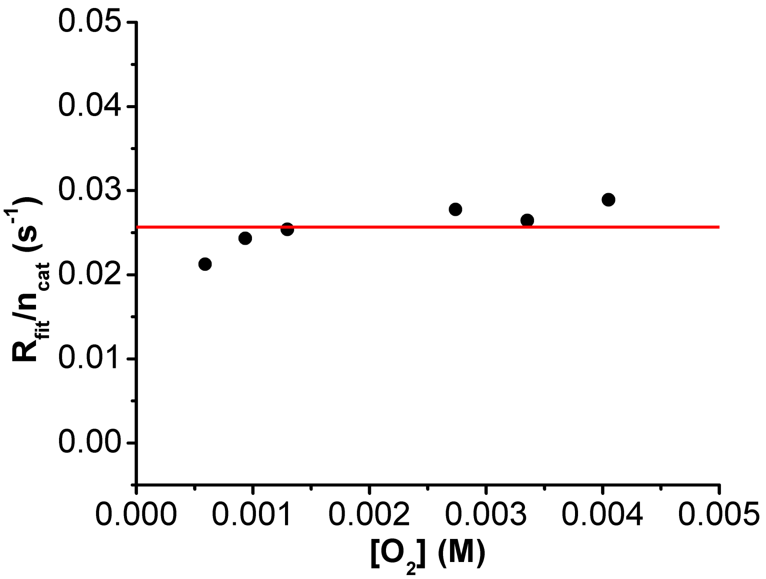

Figure S19. Calculated R_{fit}/n_{cat} values from stopped-flow spectrochemical experiments with **im**$^+$, TFAH, Cp*$_2$Fe with varying O_2 concentration. Data were fit using Kinetic Studio 4.0 (1Exp+Mx+C); n_{cat} = 2. The horizontal line represents the global average rate observed across all experiments for variable [O_2]. Concentrations: **im**$^+$ = 4 μM, TFAH = 25 mM, Cp*$_2$Fe = 1 mM.

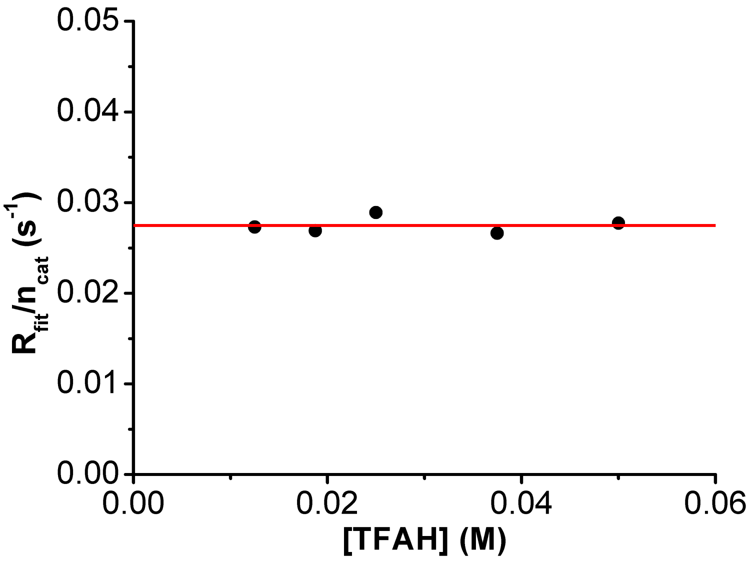

Figure S20. Calculated R_{fit}/n_{cat} values from stopped-flow spectrochemical experiments with **im**$^+$, O_2, Cp*$_2$Fe with varying TFAH concentration. Data were fit using Kinetic Studio 4.0 (1Exp+Mx+C); n_{cat} = 2. The horizontal line represents the global average rate observed across all experiments for variable [TFAH]. Concentrations: **im**$^+$ = 4 μM, O_2 = 4.05 mM, Cp*$_2$Fe = 1 mM.

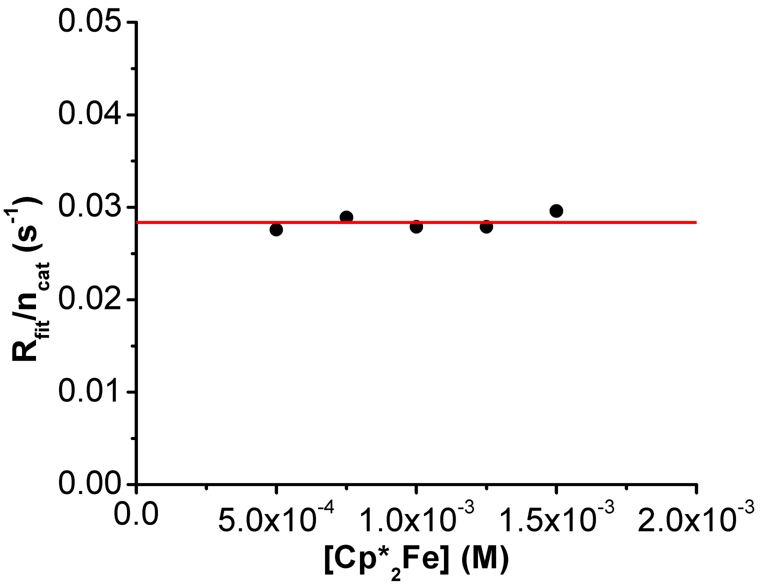

Figure S21. Calculated R_{fit}/n_{cat} values from stopped-flow spectrochemical experiments with **im$^+$**, TFAH, O_2 with varying Cp^*_2Fe concentration. Data were fit using Kinetic Studio 4.0 (1Exp+Mx+C); n_{cat} = 2. The horizontal line represents the global average rate observed across all experiments for variable [Cp^*_2Fe]. Concentrations: **im$^+$** = 4 μM, TFAH = 25 mM, O_2 = 4.05 mM.

Spectrochemical Selectivity Determination

Generally, to determine the ORR selectivity of **im$^+$**, solutions containing 8 μM [**im$^+$**] and 50 mM TFAH were sparged with O_2 gas and rapidly mixed in a 1:1 ratio with a N_2 saturated 2 mM Cp^*_2Fe solution to a final volume of 12 mL (final concentrations: 4 μM **im$^+$**, 1 mM Cp^*_2Fe, 25 mM TFAH, 4.05 mM O_2). Over the course of the reaction, 2 mL aliquots of the catalytic solution were removed and extracted with 10 mL of DCM and 5 mL of DI H_2O. The aqueous layer (3 mL) was removed and put into the cuvette and a UV-vis spectrum was taken before and after the addition of 0.1 mL of $Ti(O)SO_4$, as previously reported.[7-11] Aliquots were taken at ~15 s, ~30 s, ~1 min, and ~2 mins. Experiments were done in triplicate. A calibration curve was used to establish **Eqs S4-S5** and were used to calculate the % selectivity of H_2O_2, which was determined to be 102 ± 8.4% after 2 min.

$$O_2 + 2Cp^*{}_2Fe(1\,mM) + 2TFAH \xrightarrow{cat.[\boldsymbol{im^+}]} H_2O_2(0.5\,mM) + 2[Cp^*{}_2Fe]^+ + 2TFA^- \quad \textbf{(Eq S4)}$$

Abs@408 nm (red trace) – Abs@408 nm (black trace) = $201.2[H_2O_2]_{exp} + 0.003$

$$\frac{[\mathrm{H_2O_2}]_{\mathrm{exp}}}{0.5\ \mathrm{mM\ H_2O_2}} \times 100 = \%\ \mathrm{H_2O_2\ selectivity} \qquad \textbf{(Eq S5)}$$

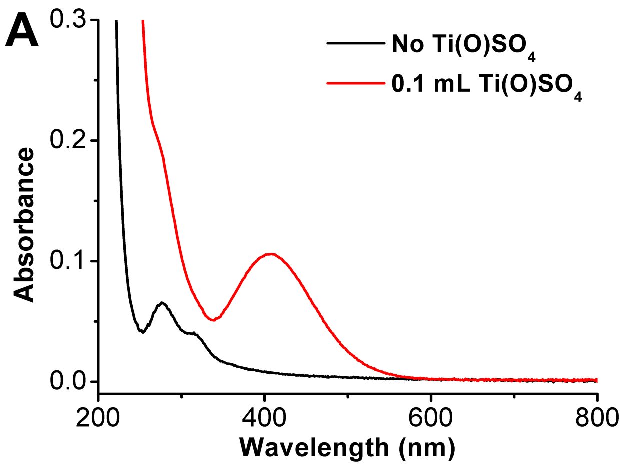

Figure S22. H_2O_2 product quantification of ORR by **im$^+$** with TFAH after 2 min. (A) UV-vis spectra of extracted solution before (black) and after (red) 0.1 mL of 0.1 M $Ti(O)SO_4$ solution was added. (B) Corrected spectra (red – black trace from A). Conditions: 4 μM **im$^+$**, 25 mM TFAH, 1 mM Cp^*_2Fe, 4.05 mM O_2 in MeCN.

Disproportionation Control

To determine the stability of H_2O_2 under catalytic conditions, control studies were conducted in the presence of **im$^+$**, TFAH, and O_2. Generally**,** solutions containing 8 μM [**im$^+$**] and 50 mM TFAH were sparged with O_2 gas and rapidly mixed in a rapidly mixed in a 1:1 ratio with a N_2 saturated urea•H_2O_2 solution (final concentrations: 4 μM **im$^+$**, 0.93 mM urea•H_2O_2, 25 mM TFAH, 4.05 mM O_2). As the solution was allowed to react, 2 mL aliquots were removed at 0 s and after 2 min, extracted with 10 mL DCM and 5 mL DI H_2O. Then, 3 mL of the aqueous layer was removed and added to the cuvette. A UV-vis spectrum was taken before and after the addition of 0.1 mL of 0.1 M $Ti(O)SO_4$ solution and the difference at 408 nm was used to determine the amount of H_2O_2 present ($[H_2O_2]_{detected}$). The % recovery was determined according to **Eq S6** from measured $[H_2O_2]_{expected}$ of the H_2O_2 stock solution. After 2 min, 104 ± 3.6 % H_2O_2 was recovered.

$$\frac{[H_2O_2]_{detected}}{[H_2O_2]_{expected}} \times 100 = \%\ H_2O_2\ recovery \qquad \textbf{(Eq S6)}$$

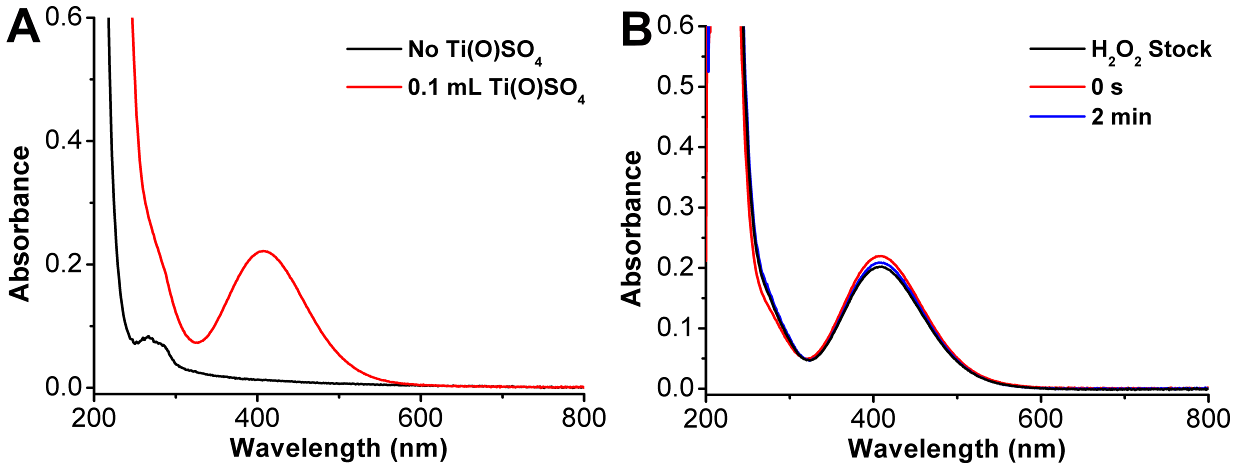

Figure S23. Stability test of urea•H_2O_2 in the presence of **im**$^+$, TFAH, and O_2 (A) before (black trace) and after (red trace) the addition of 0.1 mL of 0.1 M Ti(O)SO4 to an extracted aliquot. (B) Corrected UV-vis spectra (red – black from A) after 0 s (red) and 2 min (blue) with the H_2O_2 stock (black). Conditions: 4 μM **im**$^+$, 25 mM TFAH, 4.05 mM O_2, 0.93 mM urea•H_2O_2 in MeCN.

H_2O_2RR Control

To determine the stability of H_2O_2 in the presence of **im**^{0} and TFAH, control studies were conducted in the presence of **im**$^+$, TFAH, and Cp^*_2Fe, under an N_2 atmosphere. In a N_2-filled glovebox, urea•H_2O_2 was added to a solution containing **im**$^+$, TFAH, and Cp^*_2Fe (final concentrations: 4 μM **im**$^+$, 25 mM TFAH, 1 mM Cp^*_2Fe, and 1.5 mM urea•H_2O_2). After 2 min, a 2 mL aliquot was removed from the 'catalytic' solution and extracted with 10 mL of dry, degassed DCM and 5 mL of degassed water. Then, 3 mL of the aqueous layer was removed, and a UV-vis spectrum was taken before and after the addition of 0.1 mL of Ti(O)SO_4 solution. The difference in the absorbance at 408 nm was used to quantify the amount of H_2O_2 present according to **Eq S5**, **Eq S6** was used to calculate % H_2O_2 recovered relative to the stock H_2O_2 solution.

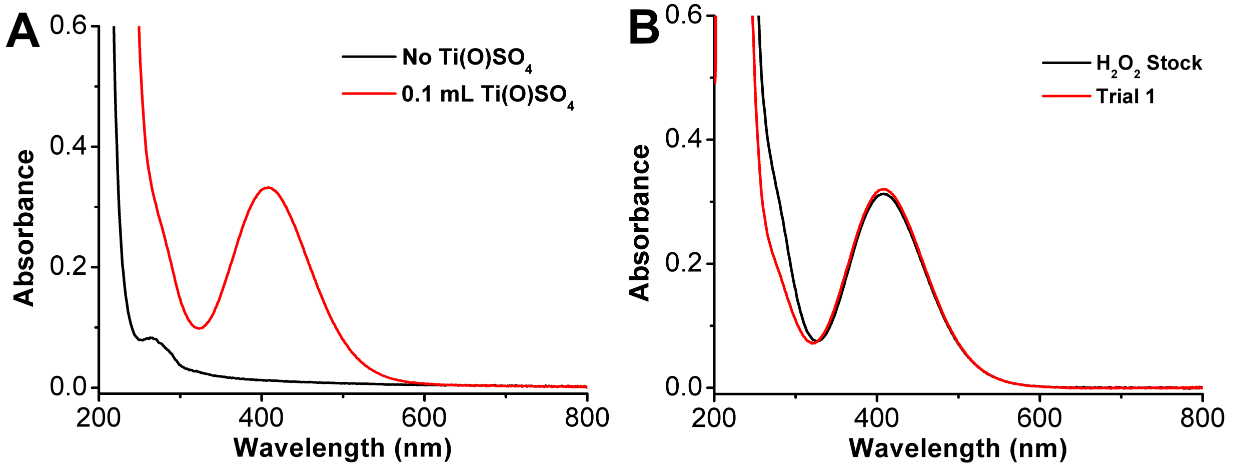

Figure S24. Stability test of urea•H_2O_2 in the presence of **im⁺**, TFAH, and Cp*₂Fe (A) before (black trace) and after (red trace) the addition of 0.1 mL of 0.1 M $Ti(O)SO_4$ to an extracted aliquot after 2 min. (B) Corrected UV-vis spectra (red – black from A) after 2 min (red) and H_2O_2 only (black). Conditions: Conditions: 4 μM **im⁺**, 25 mM TFAH, 4.05 mM O_2, 1.5 mM urea•H_2O_2 in MeCN.

Computational Methods

Geometry optimization was done with the Gaussian 16 package[12] at the B3LYP-D3(BJ)/def2-TZVP level[13-20] with a complete structural model. Dispersion and bulk solvent effects (acetonitrile = MeCN; ε = 35.688) were accounted for at the optimization stage, by using Grimme's D3 parameter set with Becke-Johnson (BJ) damping[19, 20] and the SMD continuum model,[21] respectively. The stationary points and their nature as minima (no imaginary frequencies) were characterized by vibrational analysis using the IGRRHO approach as implemented by default in the software package, which also produced enthalpy (H), entropy (S) and Gibbs energy (G) data at 298.15 K. The minima connected by a given transition state were determined by perturbing the transition states along the TS coordinate and optimizing to the nearest minimum. Free energies were corrected (ΔG_{qh}) to account for concentration effects and for errors associated with the harmonic oscillator approximation. Thus, according to Truhlars's quasi-harmonic approximation for vibrational entropy and enthalpy, all vibrational frequencies below 100 cm^{-1} were set to this value.[22] These anharmonic and concentration corrections were calculated with the Goodvibes code.[23] Concentrations were set at 0.001 M for all species unless otherwise indicated, 0.004 M for O_2, 0.500 M for TFAH, and 18.9 M for MeCN. Single point calculations for refining energy differences were completed with Orca 5.0[24] at the DLPNO-CCSD(T1)/cc-pVTZ level.[18, 25, 26] Evaluation of spin density was done at the ωB97M-D4/def2-TZVPPD level.[17, 18, 27-31] The stability of the wavefunction and spin contamination were studied at the double- and triple-zeta levels of theory. Reduction potentials from computational data were obtained according to our previous methodology by using the calculated free energy of reduction of the species of interest by [phenazine]⁻, corrected to the experimental potential of phenazine reduction vs Fc^+/Fc.[32]

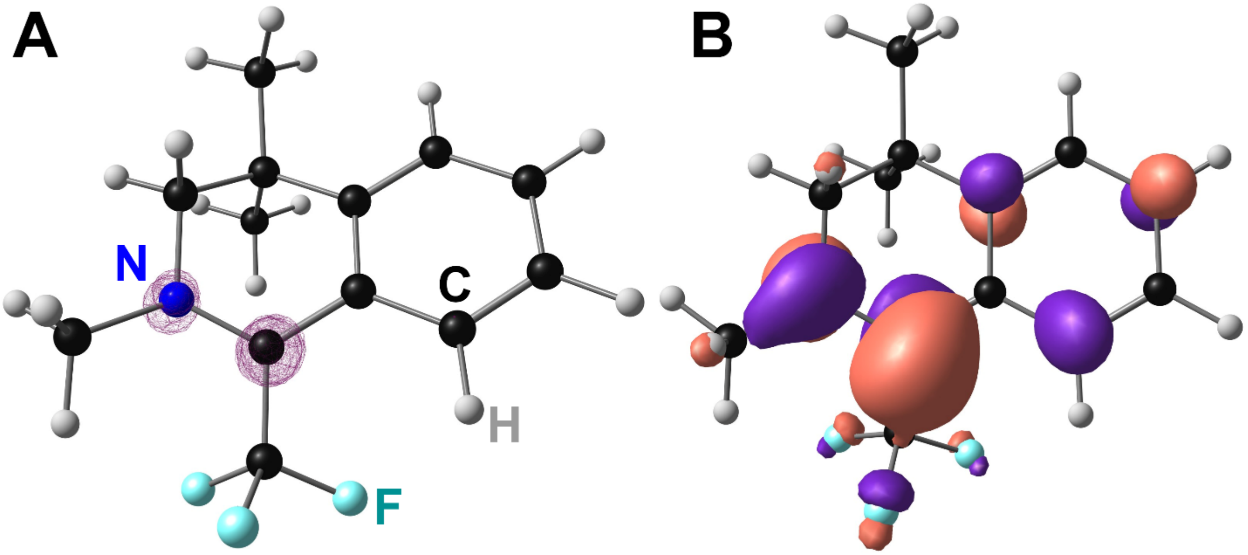

Figure S25. (A) spin density plots (0.025 iso) and (B) Kohn-Sham Orbitals (0.05 iso) of the neutral radical **im⁰** (S = ½) showing localization at C with contributions from N. Generated from the EPR calculation at the ωB97M-D4/def2-TZVPPD level of theory.

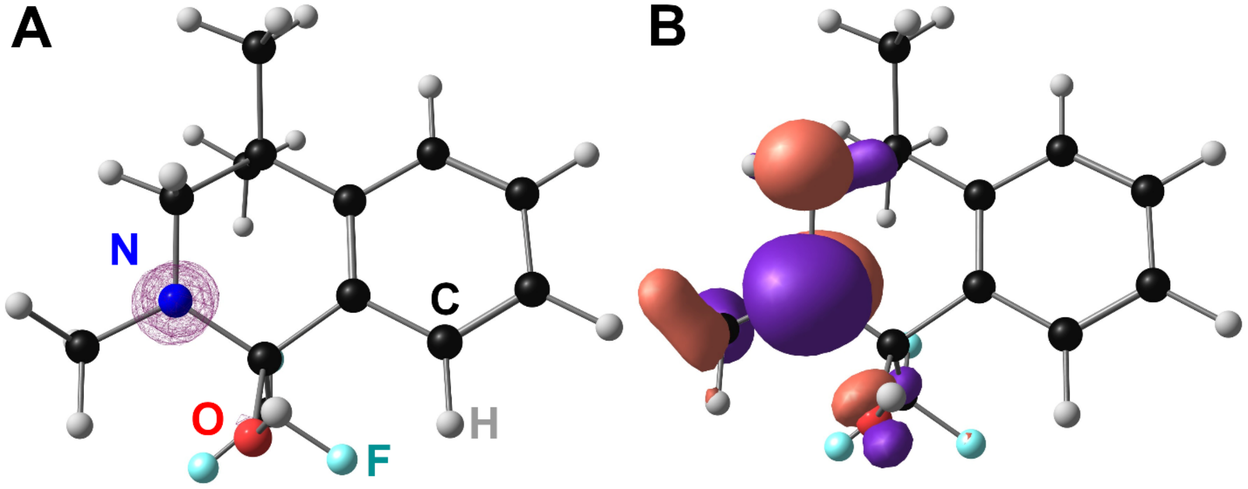

Figure S26. (A) spin density plots (0.025 iso) and (B) Kohn-Sham Orbitals (0.05 iso) of the neutral radical C(CF_3)–OH species (S = ½) showing localization at N. Generated from the EPR calculation at the ωB97M-D4/def2-TZVPPD level of theory.

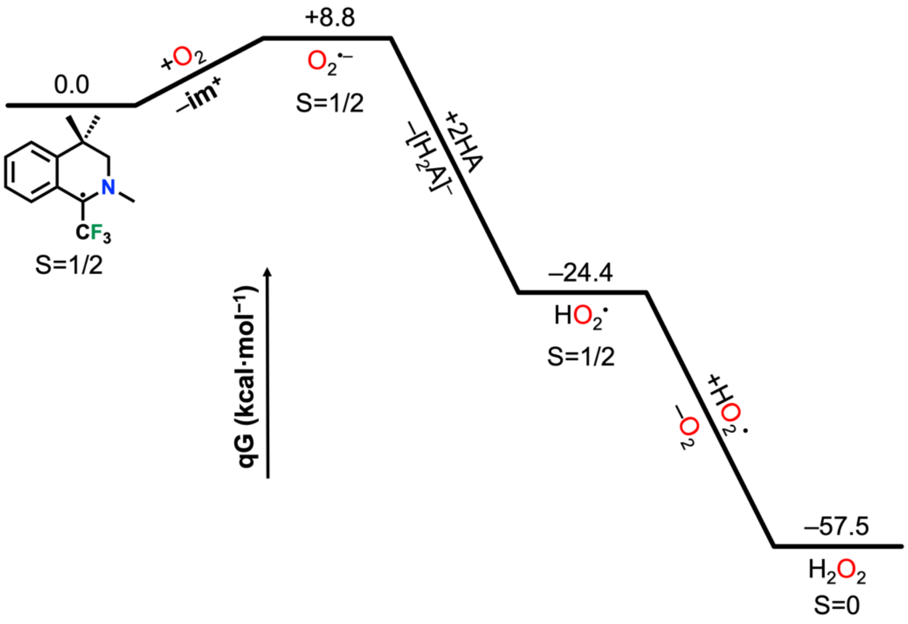

Figure S27. Thermodynamics of the catalytic cycle which produces H_2O_2 at an applied potential of –0.87 V vs Fc^+/Fc (the standard reduction potential calculated for **im⁺**). B3LYP-D3(BJ)/def2-TZVP// DLPNO-CCSD(T1)/cc-pVTZ.

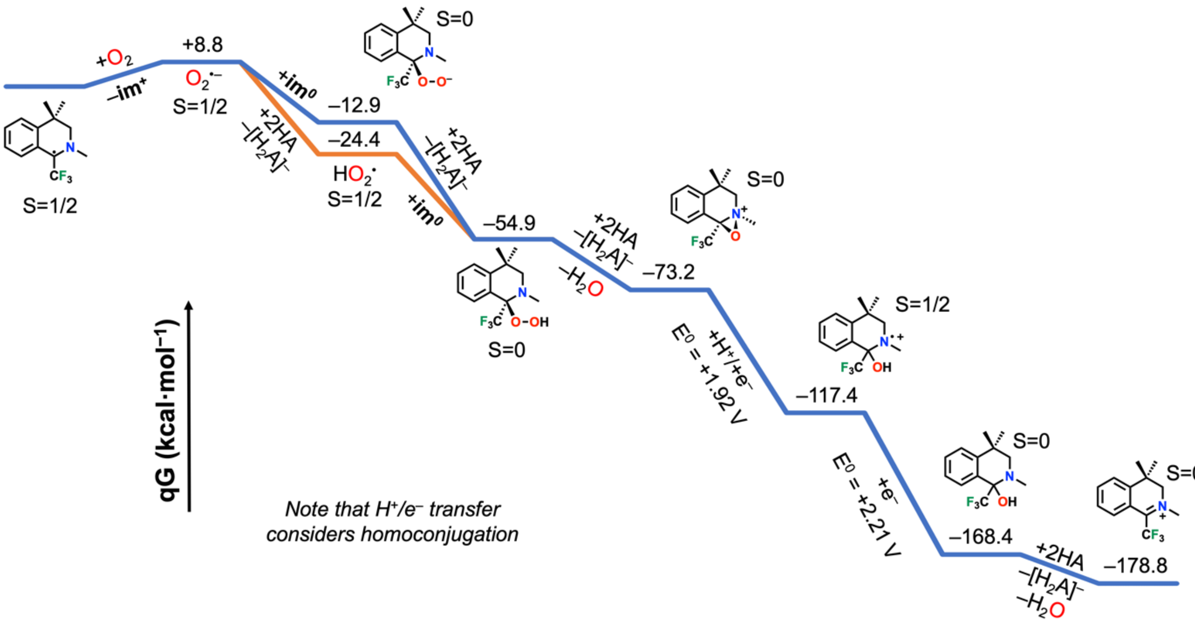

Figure S28. Thermodynamics of the catalytic cycle which produces H_2O at an applied potential of –0.87 V vs Fc^+/Fc (the standard reduction potential calculated for **im⁺**). B3LYP-D3(BJ)/def2-TZVP// DLPNO-CCSD(T1)/cc-pVTZ.

References

(1) Li, Q.; Batchelor-McAuley, C.; Lawrence, N. S.; Hartshorne, R. S.; Compton, R. G. Anomalous solubility of oxygen in acetonitrile/water mixture containing tetra-n-butylammonium perchlorate supporting electrolyte; the solubility and diffusion coefficient of oxygen in anhydrous acetonitrile and aqueous mixtures. *Journal of Electroanalytical Chemistry* **2013**, *688*, 328-335. DOI: https://doi.org/10.1016/j.jelechem.2012.07.039.

(2) Dyer, R. M. B. Advances in Organocatalytic Site Selective Oxidations. University of Virginia, Charlottesville, VA, 2023.

(3) Wang, D.; Shuler, W. G.; Pierce, C. J.; Hilinski, M. K. An Iminium Salt Organocatalyst for Selective Aliphatic C–H Hydroxylation. *Organic Letters* **2016**, *18* (15), 3826-3829. DOI: 10.1021/acs.orglett.6b01832.

(4) Costentin, C.; Savéant, J.-M. Coupling of Electrode Electron Transfers with Homogeneous Chemical Reactions. In *Elements of Molecular and Biomolecular Electrochemistry*, 2019; pp 81-181.

(5) Harraz, D. M.; Weng, S.; Surendranath, Y. Electrochemically Quantifying Oxygen Reduction Selectivity in Nonaqueous Electrolytes. *ACS Catalysis* **2023**, *13* (2), 1462-1469. DOI: 10.1021/acscatal.2c04564.

(6) Hooe, S. L.; Rheingold, A. L.; Machan, C. W. Electrocatalytic Reduction of Dioxygen to Hydrogen Peroxide by a Molecular Manganese Complex with a Bipyridine-Containing Schiff Base Ligand. *Journal of the American Chemical Society* **2018**, *140* (9), 3232-3241. DOI: 10.1021/jacs.7b09027.

(7) Cook, E. N.; Dickie, D. A.; Machan, C. W. Catalytic Reduction of Dioxygen to Water by a Bioinspired Non-Heme Iron Complex via a 2+2 Mechanism. *Journal of the American Chemical Society* **2021**, *143* (40), 16411-16418. DOI: 10.1021/jacs.1c04572.

(8) Cook, E. N.; Hooe, S. L.; Dickie, D. A.; Machan, C. W. Homogeneous Catalytic Reduction of O2 to H2O by a Terpyridine-Based FeN3O Complex. *Inorganic Chemistry* **2022**, *61* (22), 8387-8392. DOI: 10.1021/acs.inorgchem.2c00524.

(9) Hooe, S. L.; Machan, C. W. Dioxygen Reduction to Hydrogen Peroxide by a Molecular Mn Complex: Mechanistic Divergence between Homogeneous and Heterogeneous Reductants. *Journal of the American Chemical Society* **2019**, *141* (10), 4379-4387. DOI: 10.1021/jacs.8b13373.

(10) Nichols, A. W.; Cook, E. N.; Gan, Y. J.; Miedaner, P. R.; Dressel, J. M.; Dickie, D. A.; Shafaat, H. S.; Machan, C. W. Pendent Relay Enhances H2O2 Selectivity during Dioxygen Reduction Mediated by Bipyridine-Based Co–N2O2 Complexes. *Journal of the American Chemical Society* **2021**, *143* (33), 13065-13073. DOI: 10.1021/jacs.1c03381.

(11) Anson, C. W.; Stahl, S. S. Cooperative Electrocatalytic O2 Reduction Involving Co(salophen) with p-Hydroquinone as an Electron-Proton Transfer Mediator. *Journal of the American Chemical Society* **2017**, *139* (51), 18472-18475. DOI: 10.1021/jacs.7b11362.

(12) *Gaussian 16, Revision B.01*; Gaussian, Inc.: Wallingford CT, 2016. (accessed.

(13) Becke, A. D. Density-functional thermochemistry. III. The role of exact exchange. *The Journal of Chemical Physics* **1993**, *98* (7), 5648-5652. DOI: 10.1063/1.464913.

(14) Lee, C.; Yang, W.; Parr, R. G. Development of the Colle-Salvetti correlation-energy formula into a functional of the electron density. *Physical Review B* **1988**, *37* (2), 785-789.

(15) Vosko, S. H.; Wilk, L.; Nusair, M. Accurate spin-dependent electron liquid correlation energies for local spin density calculations: a critical analysis. *Canadian Journal of Physics* **1980**, *58* (8), 1200-1211. DOI: 10.1139/p80-159.

(16) Stephen, P. J.; Devlin, F. J.; Chabalowski, C. F.; Frisch, M. J. Ab Initio Calculation of Vibrational Absorption and Circular Dichroism Spectra Using Density Functional Force Fields. *The Journal of Physical Chemistry* **1994**, *98* (45), 11623-11627.

(17) Weigend, F.; Ahlrichs, R. Balanced basis sets of split valence, triple zeta valence and quadruple zeta valence quality for H to Rn: Design and assessment of accuracy. *Physical Chemistry Chemical Physics* **2005**, *7* (18), 3297-3305. DOI: 10.1039/b508541a.
(18) Weigend, F. Accurate Coulomb-fitting basis sets for H to Rn. *Physical Chemistry Chemical Physics* **2006**, *8* (9), 1057-1065. DOI: 10.1039/b515623h.
(19) Grimme, S.; Antony, J.; Ehrlich, S.; Krieg, H. A consistent and accurate ab initio parametrization of density functional dispersion correction (DFT-D) for the 94 elements H-Pu. *Journal of Chemical Physics* **2010**, *132* (15), 154104-154104. DOI: 10.1063/1.3382344.
(20) Grimme, S.; Ehrlich, S.; Goerigk, L. Effect of the damping function in dispersion corrected density functional theory. *Journal of Computational Chemistry* **2011**, *32* (7), 1456-1465. DOI: https://doi.org/10.1002/jcc.21759.
(21) Marenich, A. V.; Cramer, C. J.; Truhlar, D. G. Universal Solvation Model Based on Solute Electron Density and on a Continuum Model of the Solvent Defined by the Bulk Dielectric Constant and Atomic Surface Tensions. *J. Phys. Chem. B* **2009**, *113* (18), 6378-6396. DOI: 10.1021/jp810292n.
(22) Ribeiro, R. F.; Marenich, A. V.; Cramer, C. J.; Truhlar, D. G. Use of solution-phase vibrational frequencies in continuum models for the free energy of solvation. *Journal of Physical Chemistry B* **2011**, *115* (49), 14556-14562. DOI: 10.1021/jp205508z.
(23) *Goodvibes v.3.0.1*; 2019. (accessed.
(24) Neese, F. Software update: The ORCA program system—Version 5.0. *WIREs Computational Molecular Science* **2022**, *12* (5), e1606. DOI: https://doi.org/10.1002/wcms.1606.
(25) Dunning, T. H., Jr. Gaussian basis sets for use in correlated molecular calculations. I. The atoms boron through neon and hydrogen. *The Journal of Chemical Physics* **1989**, *90* (2), 1007-1023. DOI: 10.1063/1.456153.
(26) Weigend, F.; Köhn, A.; Hättig, C. Efficient use of the correlation consistent basis sets in resolution of the identity MP2 calculations. *The Journal of Chemical Physics* **2002**, *116* (8), 3175-3183. DOI: 10.1063/1.1445115.
(27) Mardirossian, N.; Head-Gordon, M. ωB97M-V: A combinatorially optimized, range-separated hybrid, meta-GGA density functional with VV10 nonlocal correlation. *The Journal of Chemical Physics* **2016**, *144* (21), 214110. DOI: 10.1063/1.4952647.
(28) Hellweg, A.; Hättig, C.; Höfener, S.; Klopper, W. Optimized accurate auxiliary basis sets for RI-MP2 and RI-CC2 calculations for the atoms Rb to Rn. *Theoretical Chemistry Accounts* **2007**, *117* (4), 587-597. DOI: 10.1007/s00214-007-0250-5.
(29) Caldeweyher, E.; Bannwarth, C.; Grimme, S. Extension of the D3 dispersion coefficient model. *The Journal of Chemical Physics* **2017**, *147* (3), 034112. DOI: 10.1063/1.4993215.
(30) Caldeweyher, E.; Ehlert, S.; Hansen, A.; Neugebauer, H.; Spicher, S.; Bannwarth, C.; Grimme, S. A generally applicable atomic-charge dependent London dispersion correction. *The Journal of Chemical Physics* **2019**, *150* (15), 154122. DOI: 10.1063/1.5090222 (acccessed 5/15/2023).
(31) Najibi, A.; Goerigk, L. DFT-D4 counterparts of leading meta-generalized-gradient approximation and hybrid density functionals for energetics and geometries. *Journal of Computational Chemistry* **2020**, *41* (30), 2562-2572. DOI: https://doi.org/10.1002/jcc.26411.
(32) Moreno, J. J.; Hooe, S. L.; Machan, C. W. DFT Study on the Electrocatalytic Reduction of CO2 to CO by a Molecular Chromium Complex. *Inorganic Chemistry* **2021**, *60* (6), 3635-3650. DOI: 10.1021/acs.inorgchem.0c03136.
